# *In vivo* efficacy and safety of systemically administered serinol nucleic acid-modified antisense oligonucleotides in mouse kidney

**DOI:** 10.1016/j.omtn.2024.102387

**Published:** 2024-12-18

**Authors:** Toshiki Tsuboi, Keita Hattori, Takuji Ishimoto, Kentaro Imai, Tomohito Doke, Junichiro Hagita, Jumpei Ariyoshi, Kazuhiro Furuhashi, Noritoshi Kato, Yasuhiko Ito, Yukiko Kamiya, Hiroyuki Asanuma, Shoichi Maruyama

**Affiliations:** 1 Department of Nephrology, Nagoya University Graduate School of Medicine, Nagoya, Japan; 2 Department of Nephrology, Yokkaichi Municipal Hospital, Yokkaichi, Japan; 3 Department of Nephrology and Rheumatology, Aichi Medical University, Nagakute, Japan; 4 Department of Biomolecular Engineering, Nagoya University Graduate School of Engineering, Nagoya, Japan; 5 Laboratory of Bioanalytical Chemistry, Kobe Pharmaceutical University, Kobe, Japan

**Keywords:** MT: Oligonucleotides: Therapies and Applications, antisense oligonucleotide, ASO, serinol nucleic acid, SNA, serinol nucleic acid-modified antisense oligonucleotide, SNA-ASO, nucleic acid therapeutics, kidney, SGLT2

## Abstract

Nucleic acid medicine encompassing antisense oligonucleotides (ASOs) has garnered interest as a potential avenue for next-generation therapeutics. However, their therapeutic application has been constrained by challenges such as instability, off-target effects, delivery issues, and immunogenic responses. Furthermore, their practical utility in treating kidney diseases remains unrealized. Recently, we developed a serinol nucleic acid-modified ASO (SNA-ASO) that exhibits significant nuclease resistance. In this study, we evaluated the *in vivo* efficacy of SNA-ASOs in mouse kidney. We subcutaneously administered various types of phosphorothioate-modified gapmer ASOs with SNA or 2′-*O*-methoxyethyl (2′-MOE) modifications (MOE-ASO) targeting sodium glucose cotransporter 2 (SGLT2) in mice. The subcutaneous administration of SGLT2-SNA-ASO led to a dose-dependent reduction in renal SGLT2 expression and subsequent glucosuria. The inhibitory effects of SGLT2-SNA-ASO were more potent and prolonged than those of ASOs without SNA. Moreover, SGLT2-SNA-ASO did not cause severe liver damage, unlike SGLT2-MOE-ASO. The administration of Cy5-labeled-ASOs demonstrated an early increase in renal uptake, particularly in the renal proximal tubules, when modified with SNA. In conclusion, systemic administration of SGLT2-ASO modified with the artificial nucleic acid SNA effectively suppressed renal SGLT2 expression and induced urinary glucose excretion. These results suggest that SNA-modified ASOs show potential for application in developing nucleic acid therapeutics.

## Graphical abstract

**Figure:**
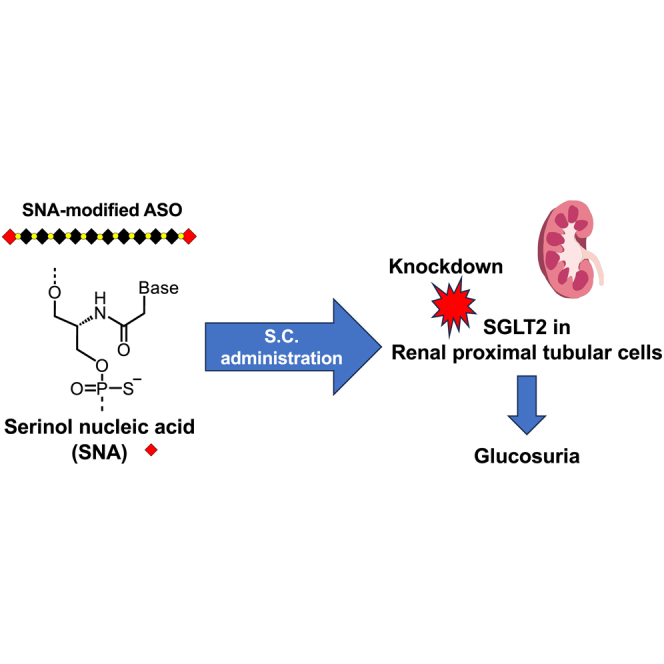

## Teaser

Ishimoto and colleagues developed a novel nucleic acid drug, serinol nucleic acid-modified antisense oligonucleotides (SNA-ASO), and tested its effectiveness in mouse kidneys. SNA-ASOs targeting SGLT2 (sodium glucose cotransporter 2) effectively reduced renal SGLT2 expression leading to glucosuria, without severe liver and kidney damage, offering potential for future therapies.

## Introduction

The dynamic landscape of nucleic acid medicine presents numerous opportunities for the development of innovative treatments for various diseases.^1^ Among these, antisense oligonucleotides (ASOs) have gained significant attention because of their potential to regulate gene expression at the post-transcriptional level.^2^ ASOs are short, synthetic, single-stranded oligonucleotides that bind to specific RNA sequences, modulating protein expression through several distinct mechanisms, including the inhibition of translation and exon skipping.^3^ This targeted regulation of gene expression has shown promise in the treatment of diseases with genetic causes ranging from monogenic disorders to cancers.^4^

Despite these promising features, the therapeutic application of ASOs has been limited by numerous challenges, such as instability, off-target effects, delivery issues, and immunogenic responses.^5^ Stability is a particularly crucial factor because unmodified ASOs are prone to rapid degradation by endonucleases and exonucleases, which can significantly reduce their therapeutic half-lives.^6^ To address these challenges, researchers have explored a variety of chemical modifications. For instance, 2′-*O*-methoxyethyl (2′-MOE) and locked nucleic acid (LNA) modifications have shown promise in improving ASO stability and binding affinity to mRNA targets.^7^^,^^8^ Phosphorothioate (PS) backbone modifications protect the ASO from degradation by nucleases.^9^ However, these modifications have drawbacks such as potential hepatotoxicity and eliciting immune responses.^10^

Several ASO-based therapies, such as nusinersen for spinal muscular atrophy and mipomersen for homozygous familial hypercholesterolemia, have been clinically applied successfully.^11^ However, ASO therapies targeting the kidney remain largely underexplored despite the potential of the organ as a therapeutic target for various renal diseases. According to previous reports, systemically administered artificial nucleic acids strongly accumulate in the kidney and liver,^6^ suggesting their potential to effectively target the kidney. Nevertheless, no nucleic acid drugs that specifically target this organ are available on the market.^12^

Recently, we developed an artificial nucleic acid known as serinol nucleic acid (SNA), which is characterized by its unique acyclic structure, low synthesis cost, and ability to form stable duplexes with cDNA/cRNA strands.^13^^,^^14^ Applications of SNA have been realized based on hybridization with RNA such as a high-sensitive molecular beacon.^15^^,^^16^ In addition, we have demonstrated efficacy of SNA-modified small interfering RNA and ASOs consisting of SNA *in vitro* studies.^17^^,^^18^ However, studies on the effects of systemic administration of SNA-modified nucleic acid-based drug candidates are sparse, and their *in vivo* characterization and therapeutic potential remain unexplored.

This study aimed to investigate the effects of systemically administered SNA-modified ASOs (SNA-ASOs) with a specific focus on their delivery to the kidney. Sodium glucose cotransporter 2 (SGLT2) has been selected as the target molecule for the evaluation of the efficacy of systemically administered SNA-ASOs in proximal tubules because of its specific expression in the luminal membrane of the S1 and S2 segments of the renal proximal tubule,^19^ ease of activity verification via urine glucose levels,^20^ and ample supporting data of ASO with 2′-MOE modifications targeting human and murine SGLT2, including sequence information from previous clinical studies.^21^^,^^22^^,^^23^

In this report, we present a novel method for modifying ASOs to enhance their stability and efficacy while minimizing potential side effects. We believe that our findings would expand the potential of ASOs in therapeutic applications and facilitate the development of nucleic acid medicines that target the kidney.

## Results

### Effects of SGLT2-ASOs on SGLT2 expression in HK-2 cells

**Figure 1:**
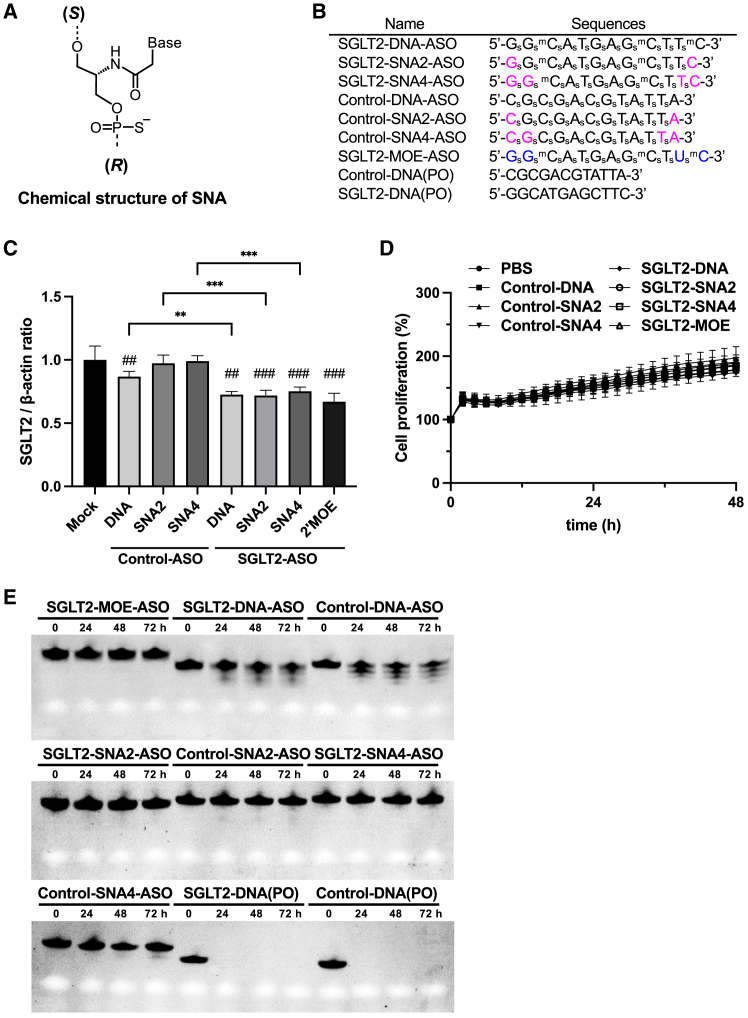
Effects of DNA-antisense oligonucleotides, serinol nucleic acid 2-ASO and 4-ASO, and 2′-methoxyethyl-ASO on sodium glucose cotransporter 2 -mRNA expression in the immortalized human proximal tubule epithelial cells (A) The chemical structure of serinol nucleic acid (SNA). In analogy to the 5′-3′ designation for DNA, the (S)-(R) designation for SNA based on the configuration at C2′ was used. (B) Sequences and structures of various antisense oligonucleotides (ASOs). Magenta letters indicate SNA, black letters indicate DNA, and blue letters indicate the 2′-methoxyethyl (2′-MOE) modification. ^m^C, 5-methyl cytosine; subscript s, PS. (C) Human proximal tubule epithelial cells (HK-2) cells were transfected with control ASOs and sodium glucose cotransporter 2 (SGLT2)-ASOs for 24 h. Quantitative PCR (qPCR) analysis of SGLT2 mRNA expression (*n* = 6). β-Actin was used as the internal control. ∗*p* < 0.05; ∗∗*p* < 0.01; ∗∗∗*p* < 0.001. (D) HK-2 cells were transfected with control ASOs and SGLT2-ASOs, and imaged every 4 h for up to 48 h (*n* = 3). The cell proliferation rate is plotted graphically, with 0 h as 100%. (E) Effect of modifications on ASO degradation in 50% fetal bovine serum (FBS). Each ASO was incubated with 50% FBS at 37°C. Aliquots taken at the indicated times of incubation were analyzed by 20% denaturing PAGE. Data represent means ± SEMs.

The chemical structure of SNA is shown in Figure 1A. The ASOs used in this study were gapmer-type ASOs in which the DNA region was located in the center and an artificial nucleic acid was introduced at both ends.^2^ Therefore, ASOs were expected to allow RNase H1-mediated degradation of the target mRNA through recognition of RNA/DNA heteroduplex by RNase H1. To verify the sequence-specific knockdown of SGLT2 by SNA-ASOs, PS-modified DNA-ASO, SNA-modified gapmer ASOs (SNA2-ASO and SNA4-ASO contain one and two SNA modifications at each end, respectively), and 2′-MOE-modified gapmer ASO (MOE-ASO) and respective control-ASOs (Figure 1B) were tested in HK-2 cells (immortalized human proximal epithelial cells). The sequence of SGLT2-ASOs was identical to that of SGLT2-MOE-ASO, which targets both human and mouse SGLT2 (Data S1), as evidenced in previous clinical investigations.^21^^,^^22^^,^^23^ As shown in Figure 1C, control-SNA-ASOs (control-SNA2-ASO or control-SNA4-ASO) did not exert any effect on SGLT2 expression in contrast to that observed in mock-transfected cells. As reported previously,^21^^,^^22^^,^^23^ SGLT2-MOE-ASO showed significant inhibitory effects on SGLT2 expression (Figure 1C). In addition, SGLT2-SNA2-ASO and SGLT2-SNA4-ASO significantly suppressed SGLT2 expression compared with mock transfection and respective control-SNA-ASOs (Figure 1C). No differences in cell proliferation or morphology were observed between the groups (Figures 1D and S1). Moreover, the protein levels of caspase-3 and cleaved-caspase-3 were not elevated in SNA-ASOs or MOE-ASO groups (Figure S2). Next, to examine the stability against nucleases of SNA-ASOs, ASOs with various chemical modifications were incubated in 50% fetal bovine serum (FBS). As shown in Figure 1E, DNA-ASOs without any modification (PO) degraded completely in 50% FBS within 24 h. However, PS-modified ASOs (DNA-ASOs) exhibited enhanced resistance to degradation. Furthermore, similar to MOE-ASO, both SNA2-ASOs and SNA4-ASOs remained intact (Figure 1E). These results indicate that SNA-ASOs have sequence-specific inhibitory effects, are stable against serum, and exert no apparent influence on cell morphology and proliferation.

### SGLT2-SNA-ASOs suppressed renal SGLT2 expression in a dose-dependent manner

**Figure 2:**
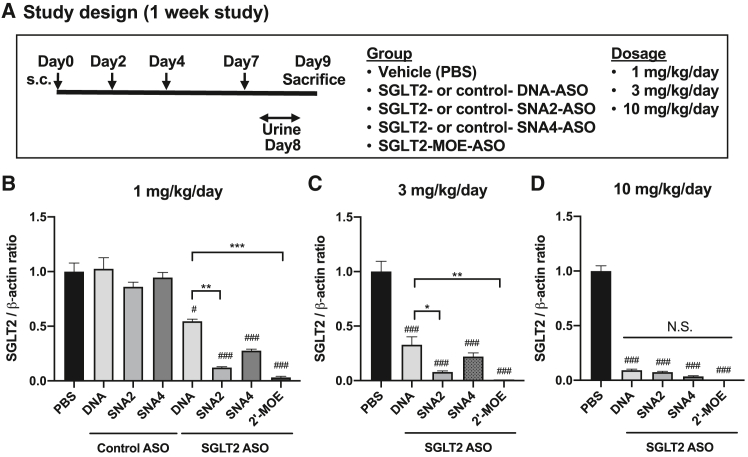
SGLT2-SNA-ASOs suppress renal SGLT2 expression in a dose-dependent manner SGLT2-ASOs and control-ASOs were subcutaneously (s.c.) administered to mice at doses of 1, 3, and 10 mg/kg/day thrice per week for 1 week. (A) Study design. (B–D) qPCR analysis of SGLT2 expression in the kidney (*n* = 4). β-Actin was used as the internal control. Data represent means ± SEMs. #*p* < 0.05; ##*p* < 0.01; ###*p* < 0.001 vs. respective control and PBS. ∗*p* < 0.05; ∗∗*p* < 0.01; ∗∗∗*p* < 0.001; N.S., not significant.

**Figure 3:**
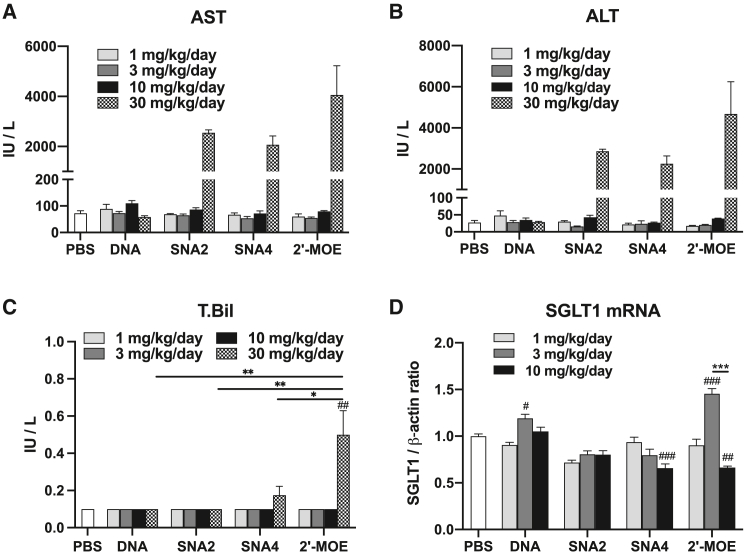
Adverse effects of SGLT2-ASOs SGLT2-ASOs were subcutaneously administered to mice at doses of 1, 3, 10, and 30 mg/kg/day thrice per week for 1 week. (A) Serum aspartate aminotransferase (AST) levels (*n* = 4). (B) Serum alanine aminotransferase (ALT) levels (*n* = 4). (C) Total bilirubin levels (*n* = 4). (D) qPCR analysis of SGLT1 expression in the kidneys (*n* = 4). β-Actin was used as the internal control. Data are presented as the means ± SEMs. #*p* < 0.05; ##*p* < 0.01; ###*p* < 0.001 vs. respective 1 mg/kg group; ∗∗∗*p* < 0.001.

Next, we evaluated the sequence-specific inhibitory effect of SGLT2-DNA-ASO, SGLT2-SNA ASOs, and SGLT2-MOE-ASO on *in vivo* SGLT2 mRNA expression. SGLT2-ASOs and control-ASOs were subcutaneously administered at escalating doses to mice for 1 week (Figure 2A), and the expression levels of SGLT2 mRNA in mouse kidneys were evaluated using quantitative PCR (qPCR) (Figures 2B–2D and S3). As shown in Figure 2B, all SGLT2-ASOs (including SGLT-2-SNA2-ASO and SGLT-2-SNA4 ASO) administered at a dose of 1 mg/kg/day significantly suppressed SGLT2 mRNA expression compared with the respective control-ASO groups. Notably, SGLT2-SNA2-ASO administered at 1 and 3 mg/kg/day significantly reduced SGLT2 expression compared with SGLT2-DNA-ASO (Figures 2B and 2C). Furthermore, the inhibitory effects exerted by SGLT2-SNA-ASOs were dependent on the SGLT2-SNA-ASO dosage (1, 3, and 10 mg/kg/day; Figures 2B–2D and S3). Thus, SNA introduced at the terminal sides of the ASO improved ASO activity compared with DNA-ASO. These inhibitory effects were most potent in the SGLT2-MOE-ASO group. Next, we examined the adverse effects of SGLT2-ASOs by further increasing the dosage to 30 mg/kg/day. Although no apparent treatment-associated physical changes, mortality, body weight change, hypoproteinemia, or hypoalbuminemia were observed in treated mice (Figure S4), laboratory examination revealed that a maximum dose of 30 mg/kg/day of SGLT2-ASOs induced very severe liver damage, as indicated by increased blood levels of liver enzymes such as aspartate aminotransferase (AST), alanine aminotransferase (ALT) (AST and ALT >2,000 IU/L; Figures 3A and 3B). Furthermore, only mice administered 30 mg/kg/day SGLT2-MOE-ASO showed a significant increase in total bilirubin levels (Figure 3C). However, no significant liver dysfunction was observed in mice administered SGLT2-ASOs at doses of 1, 3, or 10 mg/kg/day (Figures 3A–3C). Mild inflammatory cell infiltration and mild fibrosis were observed in the livers of mice administered 30 mg/kg/day SNA-ASOs or MOE-ASO (Figures S5 and S6). In addition, mild and slight interstitial fibrosis and tubular atrophy were detected in the kidneys of mice administered 30 mg/kg/day MOE-ASO or SNA4-ASO, respectively (Figures S7 and S8). Hence, a dosage of 10 mg/kg/day was used in subsequent experiments. To determine whether ASOs could induce the sequence-specific knockdown of SGLT2, we analyzed another sodium glucose cotransporter 1 (*SGLT1*), an SGLT subtype commonly expressed in the proximal tubules. None of the SGLT2-ASOs showed any apparent dose-dependent suppression of SGLT1 mRNA expression (Figure 3D). These results indicate that systemically administered SGLT2-SNA-ASOs suppress renal SGLT2 expression in a dose-dependent and sequence-specific manner.

### SGLT2-SNA2-ASOs exhibited good efficacy and low toxicity in mouse kidney and liver

**Figure 4:**
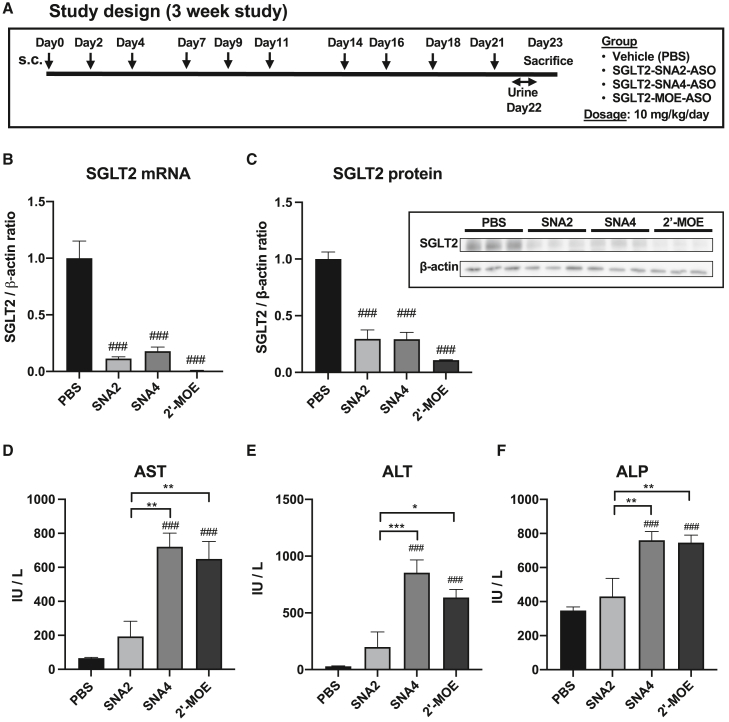
Administration of various SGLT2-ASOs for 3 weeks in mice SGLT2-ASOs were subcutaneously administered to mice at doses of 10 mg/kg/day thrice per week for 3 weeks. (A) Study design. (B) qPCR analysis of SGLT2 expression in kidneys (*n* = 4–8). (C) Western blotting analysis of SGLT2 expression in the kidneys (*n* = 3). β-Actin was used as the internal control. (D) Serum AST levels (*n* = 4–8). (E) Serum ALT levels (*n* = 4–8). (F) Serum alkaline phosphatase (ALP) levels (*n* = 4–8). Data are presented as the means ± SEMs. #*p* < 0.05; ##*p* < 0.01; ###*p* < 0.001 vs. PBS; ∗*p* < 0.05; ∗∗*p* < 0.01; ∗∗∗*p* < 0.001.

To evaluate the long-term efficacy and safety of the SNA-ASOs, SGLT2-SNA2-ASO, SGLT2-SNA4-ASO, and SGLT2-MOE-ASO were administered subcutaneously to mice for 3 weeks (3 times per week, 10 times in total) at a dose of 10 mg/kg/day (Figure 4A). SGLT2-SNA-ASOs and SGLT2-MOE-ASO significantly reduced mRNA and protein levels of SGLT2 in the kidney (Figures 4B and 4C). Based on these data, SGLT2-SNA2-ASO exhibited high and comparable knockdown activity to that of SGLT2-SNA4-ASO. Notably, liver dysfunction was significantly milder in mice administered SGLT2-SNA2-ASO than in those administered SGLT2-SNA4-ASO or SGLT2-MOE-ASO (Figures 4D–4F).

### SGLT2-SNA2-ASO suppressed renal SGLT2 expression without severe liver and renal tubular damage

**Figure 5:**
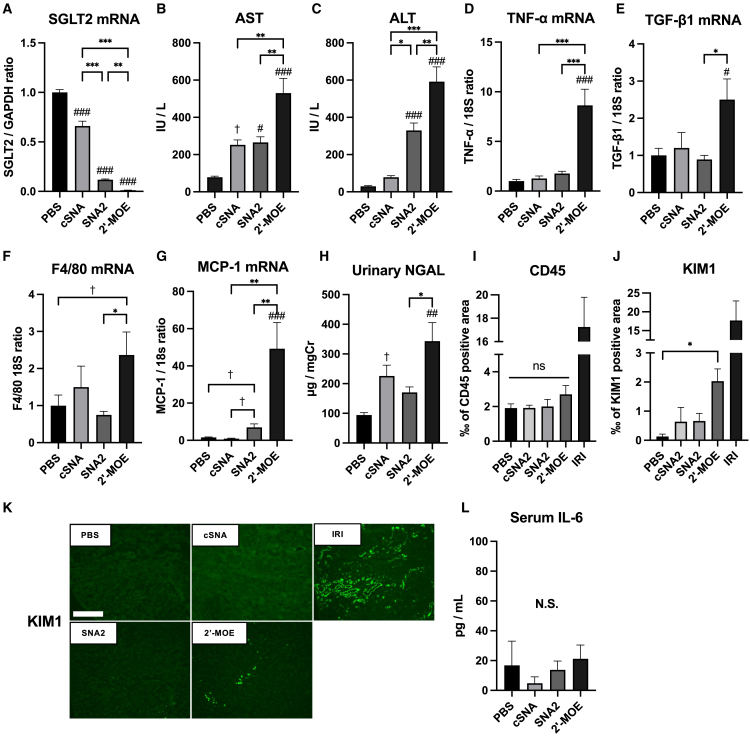
SGLT2-SNA2-ASO suppressed renal SGLT2 expression without severe liver damage SGLT2-SNA2-ASO, SNA-modified control-ASO (cSNA-ASO) or SGLT2-MOE-ASO were subcutaneously administered to mice at doses of 10 mg/kg/day thrice per week for 3 weeks (A) qPCR analysis of SGLT2 expression in the kidneys (*n* = 8). (B) Serum AST levels (*n* = 4–8). (C) Serum ALT levels (*n* = 4–8). (D–G) qPCR analysis for detecting TNF-α (D), TGF-β1 (E), F4/80 (F), and MCP-1 (G) expression in the liver (*n* = 8). We used 18S as the internal control. (H) Urinary NGAL levels (*n* = 8). (I) Quantification of CD45 positive area in kidneys (*n* = 4). (J and K) Representative images and quantification of KIM-1^+^ area in kidneys (*n* = 4). Kidney specimens from renal ischemia-reperfusion injury (IRI) were used as a positive control for KIM-1 staining. Scale bar, 500 μm. (L) Serum IL-6 levels (*n* = 4). Data are presented as the means ± SEMs. #*p* < 0.05; ##*p* < 0.01; ###*p* < 0.001 vs. PBS; ∗*p* < 0.05; ∗∗*p* < 0.01; ∗∗∗*p* < 0.001; †*p* < 0.05 by post hoc Tukey’s multiple comparisons after one-way ANOVA, with the exception of the MOE-ASO group.

To evaluate the hepatotoxicity of SNA-ASOs and confirm that it is lower than that of MOE-ASO, a second experiment was performed, in which SGLT2-SNA2-ASO and SGLT2-MOE-ASO (*n* = 8 mice per group) were administered for a 3-week period. An all-SNA-modified control-ASO (cSNA-ASO) was used as a representative control for SNA-ASOs, also serving the purpose of evaluating the toxicity of SNA modifications. Significant suppression of renal SGLT2 mRNA expression was observed in both SGLT2-SNA2-ASO- and SGLT2-MOE-ASO-administered mice (Figure 5A). Urinary glucose excretion was highly increased in both SGLT2-SNA2-ASO- and SGLT2-MOE-ASO-administered mice but was higher in mice administered SGLT2-MOE-ASO owing to its association with SGLT2 mRNA suppression (Table S1). No significant differences were observed in blood glucose levels, body weight, or liver weight between the groups (Table S1). However, liver dysfunction was observed in both groups, and it was significantly more severe in the MOE-ASO group than in the SGLT2-SNA2-ASO and cSNA-ASO groups (Figures 5B and 5C). Liver tissues derived from the SGLT2-MOE-ASO group demonstrated increased expression of tumor necrosis factor (TNF)-α, transforming growth factor (TGF)-β1, and the murine monocyte-macrophage marker F4/80 with mild liver fibrosis when compared with those from the SGLT-2-SNA2-ASO group (Figures 5D–5F and S9). Although hepatic monocyte chemoattractant protein-1 (MCP-1) was also elevated in the SGLT2-SNA2-ASO group, the SGLT-2-MOE-ASO group showed a significant increase in MCP-1 levels, which was associated with the severity of transaminitis (Figure 5G). Regarding the kidneys, the urinary neutrophil gelatinase-associated lipocalin (NGAL) level was significantly elevated more in mice administered SGLT2-MOE-ASO than in those administered SGLT2-SNA2-ASO (Figure 5H). In the kidneys, CD45^+^ cells tended to be more abundant in the SGLT2-MOE-ASO group, and the KIM-1^+^ area significantly increased in the MOE-ASO group, although the SNA-ASO groups also showed slight increases in KIM-1 (Figures 5I–5K and S10). KIM-1 is primarily expressed in the proximal tubules of the kidney and is widely recognized as a marker of proximal tubule injury and stress. Kidneys from murine ischemia-reperfusion injury (IRI) models were used as positive controls for both CD45 staining and KIM-1 staining, highlighting the infiltration of immune cells and the expression of KIM-1 in damaged proximal tubules. Moreover, mild interstitial fibrosis and tubular atrophy were detected in the kidneys of mice administered 10 mg/kg/day MOE-ASO for 3 weeks, respectively (Figure S11). There were no significant differences in kidney weight and serum creatinine or interleukin (IL)-6 levels (Figure 5L; Table S1). These data indicated that SNA2-ASO was safer than MOE-ASO in terms of adverse hepatic and renal events.

### SNA modification prolonged the knockdown effects by ASO

**Figure 6:**
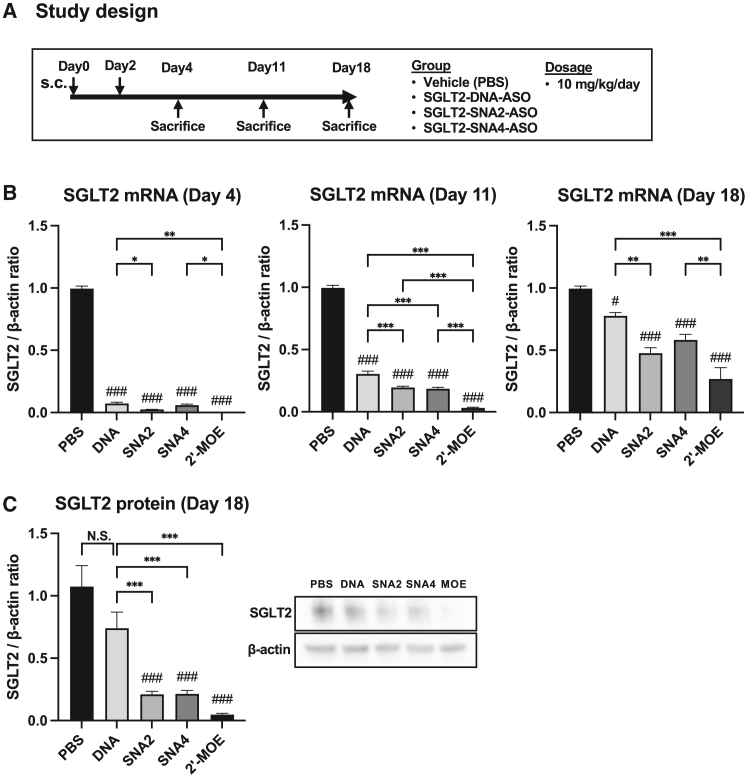
Evaluation of the *in vivo* stability of systemically administered SNA-ASOs SGLT2-DNA-ASO, SGLT2-SNA2-ASO, and SGLT2-SNA4-ASO were subcutaneously administered to the mice twice at a dose of 10 mg/kg/day (A) Study design. (B) qPCR analysis of SGLT2 expression in the kidney on days 4, 11, and 18 (*n* = 8). (C) Western blot analysis of SGLT2 protein in kidney samples on day 18 (*n* = 6). β-Actin was used as the internal control. Data are presented as the means ± SEMs. #*p* < 0.05; ###*p* < 0.001 vs. PBS; ∗*p* < 0.05; ∗∗*p* < 0.01; ∗∗∗*p* < 0.001; N.S., not significant.

To investigate the *in vivo* duration of the knockdown effects of SNA-ASO, we administered SGLT2-DNA-ASO, SGLT2-SNA2-ASO, SGLT2-SNA4-ASO, and SGLT2-MOE-ASO to mice twice, with each dose (10 mg/kg/day) administered every 2 days at a dose of 10 mg/kg/day, without any additional doses. We examined the expression levels of renal SGLT2 mRNA on days 4, 11, and 18 (Figure 6A). All SGLT2-ASOs significantly suppressed renal SGLT2 expressions on day 4, with SNA2-ASO and MOE-ASO identified to exert the most potent inhibitory effects. However, the rapid decrease in SGLT2 knockdown efficacy was observed in the SGLT2-DNA-ASO group compared with that in the SGLT2-SNA-ASO groups and the SGLT2-MOE ASO group (Figures 6B and 6C), indicating that SNA modification, similar to MOE modification, prolonged the SGLT2-ASO-mediated suppression of SGLT2 mRNA.

### Evaluation of *in vivo* distribution of systemically administered ASOs

**Figure 7:**
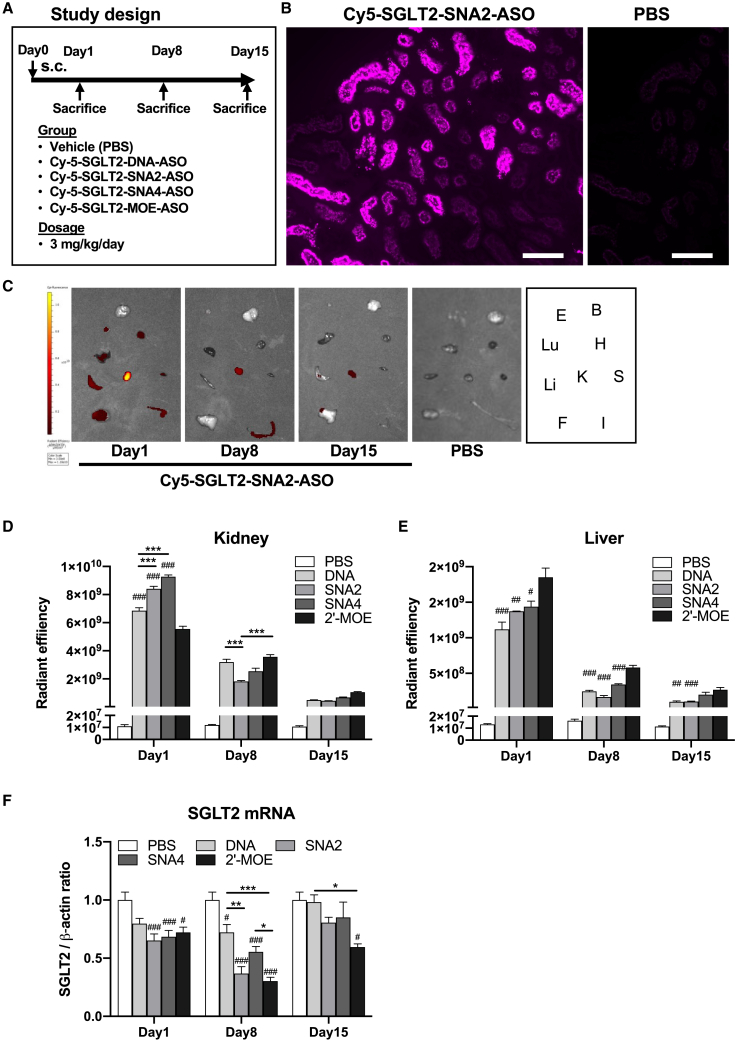
Evaluation of the *in vivo* distribution of systemically administered various ASOs Cy-5-labeled SGLT2-DNA-ASO, SGLT2-SNA2-ASO, SGLT2-SNA4-ASO, SGLT2-MOE-ASO, or PBS (vehicle control) were subcutaneously administered to mice as a single dose of 3 mg/kg (A) Study design. (B) Representative fluorescence microscopy images of kidneys from mice 24 h after the administration of Cy-5 labeled SNA2-ASO or PBS. Scale bar, 100 μm. (C) Representative images of fluorescence intensity in each organ, including the kidney (K), liver (Li), brain (B), lung (Lu), heart (H), intestine (I), eye (E), spleen (S), and epididymal fat (F), on days 1, 8, and 15 after ASO or PBS administration. Right: layout of each tissue. (D and E) Fluorescence intensities in the kidney (D) and liver (E) on days 1, 8, and 15 after ASO administration (*n* = 4). (F) qPCR analysis of SGLT2 expression in the kidney on days 1, 8, and 15 (*n* = 4). β-Actin was used as the internal control. Data are presented as the means ± SEMs. #*p* < 0.05; ###*p* < 0.001 vs. respective PBS; ∗*p* < 0.05; ∗∗*p* < 0.01; ∗∗∗*p* < 0.001.

To evaluate the organ distribution of SNA-ASO, Cy5-labeled SGLT2-ASOs were administered to mice at a single dose of 3 mg/kg (Figure 7A). Fluorescence microscopy of kidney specimens collected 24 h after administering Cy-5-labeled SNA2-ASO or phosphate-buffered saline (PBS) revealed that SNA2-ASO was predominantly incorporated into the outer layer of the kidney, known as the renal cortex, particularly in the tubular epithelial cells, but not into the glomeruli (Figures 7B, S12, and S13). Next, the fluorescence intensities of each organ, including the kidney, liver, brain, lung, heart, intestine, eye, spleen, and epididymal fat, were examined using an *in vivo* imaging system (IVIS) on days 1, 8, and 15 after ASO administration (Figures 7C and S14). Although the fluorescence intensity declined over time, the highest fluorescence intensity was maintained in the kidney, followed by the second highest fluorescence intensity in the liver. On day 1, the fluorescence intensity in the kidney was significantly higher in the SNA-ASO group than that in the DNA-ASO and MOE-ASO groups (Figure 7D). In contrast, the fluorescence intensity in the liver on days 1, 8, and 15 was significantly higher in the MOE-ASO group than in the SNA-ASO group (Figure 7E). Prolonged knockdown of SGLT2 mRNA by SNA-ASOs and MOE-ASOs was also observed (Figure 7F).

## Discussion

We have previously reported the properties and effectiveness of SNA modifications in ASOs *in vitro*.^17^^,^^18^ However, the *in vivo* effectiveness has not been reported. Thus, it remains unclear whether SNA-ASOs function effectively within living organisms, particularly in comparison with existing nucleic acid modifications. In this study, SNA-SGLT2-ASOs administered via subcutaneous injection to mice dose dependently suppressed SGLT2 mRNA and protein expression in the kidney, which in turn promoted urinary glucose excretion. To the best of our knowledge, this is the first report demonstrating the efficacy of systemically administered SNA-ASO in individual animals.

The major finding of this study is that systemically administered SNA-modified gapmer SGLT2-ASO can specifically suppress SGLT2 mRNA and protein expression in the kidney in a dose-dependent manner (Figures 2 and 3). The loss of SGLT2 function induced by SNA-modified SGLT2-ASOs was verified by the appearance of glucosuria, which is similar to the effect exerted by SGLT2 inhibitors used in clinical practice.^20^ Notably, although SGLT2-SNA2-ASO tended to suppress SGLT2 expression similarly to SGLT2-SNA4-ASO (Figures 2 and 4), liver damage in SGLT2-SNA2-ASO-administered mice was significantly milder than in mice administered SGLT2-SNA4-ASO (Figure 4). These results suggest that the gapmer ASO with a single SNA modification on each side (SNA2-ASO) may be the optimal structure for the application of SNA in the development of nucleic acid therapy.

The second major finding is that systemic administration of the SNA modified ASO, particularly SNA2-ASO, did not induce severe liver damage compared with that induced by the 2′-MOE-modified SGLT-2 ASO (Figure 5). The 3-week SGLT2-MOE-ASO administration induced liver dysfunction with inflammation (noted by hepatic TNF-α, TGF-β1, F4/80, and MCP-1 expressions). In contrast, administration of SGLT2-SNA2-ASO resulted in only a mild increase in serum liver enzymes, and with either no or only a mild increase in inflammatory mediators in the liver. The severe liver damage observed in mice administered SGLT2-MOE-ASO might have been induced by a significantly higher accumulation of SGLT2-MOE-ASO in the liver compared with that of SGLT2-SNA-ASOs (Figure 7E). Furthermore, the urinary NGAL concentration and renal tubular KIM-1 expression, which are early detection markers of acute kidney injury, were significantly higher in mice administered SGLT2-MOE-ASO than those in mice administered SGLT2-SNA2-ASO (Figure 5), despite lower uptake by the kidneys (Figure 7D). In a clinical study, the systemic administration of gapmer ASO with 2-MOE modifications against SGLT2 induced an unexpected increase in serum creatinine level accompanied by increased urinary excretion of β-2-microglobulin and KIM-1,^23^ although the preclinical data did not suggest that 2′-MOE ASOs would pose as high of a risk of nephrotoxicity.^24^^,^^25^ These results indicate that although the effects of SGLT2-MOE-ASO on SGLT2 mRNA and urinary glucose were more potent than those of SGLT2-SNA2-ASO, the SNA modification, especially SNA2-ASO, may be a useful option in the development of ASO therapy for the kidneys from a safety perspective.

When comparing SNA-modified SGLT2-ASO and SGLT2-DNA-ASO, the suppressive effect on mRNA was maintained for a longer duration with the addition of the SNA modification (Figures 6 and 7E). This was likely attributed to the increased *in vivo* stability resulting from the SNA modification, which in turn led to an early increase in uptake by the kidneys (Figure 7D). Additionally, *ex vivo* imaging using fluorescently labeled ASOs showed the strongest fluorescence intensity in the kidneys (Figure 7C). Although fluorescence intensity decreased over time, the kidneys maintained the highest fluorescence intensity, which was consistent with the initial fluorescence intensity. These results suggest the potential therapeutic advantages of targeting kidney diseases.

This study has some limitations. We employed relatively short gapmer ASOs (12-mers) against SGLT2 as examined in prior clinical studies.^21^^,^^22^^,^^23^ Investigations using SNA-ASOs of varying lengths and structures are needed, and comprehensive analyses of off-target effects and toxicity using methods such as RNA sequencing are also necessary. It is also essential to extend this study to other molecular targets.

In conclusion, systemic administration of SGLT2-ASO modified with the artificial nucleic acid SNA effectively suppressed renal SGLT2 expression and induced urinary glucose excretion. These results suggest that SNA-ASOs can be utilized in the development of nucleic acid-based therapeutics targeting the kidneys.

## Materials and methods

### ASOs

Sequence information for the gapmer ASO-targeting human and murine SGLT2 was obtained from a previous study^21^ and shown in Data S1. This ASO is a gapmer-type 12-base PS oligonucleotide that consists of a central region of deoxyribonucleotides (gaps) flanked by SGLT2-MOE-ASOs. We also designed a negative-control ASO (control ASO). Using these sequences, we generated several types of gapmer ASOs with two or four SNA modifications on both sides (SNA2-ASO and SNA4-ASO) and ASOs without 2′-MOE or SNA modifications (DNA-ASO). The ASOs were tested in cell culture studies, and the sequences and structures of the ASOs are listed in Figure 1B. For the *in vivo* study, another PS cSNA-ASO, in which each nucleotide contains an SNA modification, was also designed: 5′-TATGATGTCCATGTCGTACGC-3′. Cy5-labeled SGLT2-ASOs were also generated. All ASOs were synthesized by Nicca Chemical and Hokkaido System Science.

### ASO degradation assay in FBS

ASO solutions (20 μM) in 50% FBS solution (total 10 μL) were incubated at 37°C. Aliquots were removed at indicated times and were added to a loading buffer containing ethylenediaminetetraacetic acid (11 mM) and bromophenol blue (0.015%). Aliquots were subjected to electrophoresis on 20% polyacrylamide gels containing 8 M urea at 300 V for 120 min. Gels were analyzed using ImageQuant LAS 4000 (Cytiva), with bands stained with SYBR Gold Nucleic Acid Gel Stain (Thermo Fisher Scientific).

### Cell culture and transfection

Immortalized human renal proximal tubular epithelial cells (HK-2) were obtained from the American Type Culture Collection. All cells were maintained in Dulbecco’s modified Eagle’s medium and Ham’s F-12 medium (DMEM/F12, Gibco) supplemented with 10% heat-inactivated FBS (Sigma) and 1% penicillin-streptomycin (Gibco). Cells were grown in a humidified atmosphere with 5% CO_2_ at 37°C. The cells were plated at a density of 2.0–3.0 × 10^5^ cells/well onto a 6-well plate with 2.5 mL DMEM/F12 media containing 10% FBS and incubated for 24 h until 80%–90% confluency was achieved. Next, 50 nM SGLT2-ASOs or control-ASOs were transfected with Lipofectamine 3000 (Thermo Fisher Scientific). After 24 h of transfection, the cells were collected and analyzed. Apoptotic responses were evaluated by western blotting for caspase-3 and cleaved caspase-3. Lipopolysaccharide (10 μg/mL, Sigma-Aldrich) was used as a positive control. In the cell proliferation experiment, the cells were plated at a density of 1.0×10^5^ cells/well in a 12-well plate and incubated for 24 h until 50% confluency was achieved. Then, 50 nM SGLT2-ASOs and control-ASOs were transfected using Lipofectamine 3000. Images were captured every 4 h for 48 h, and the cell confluence rate was measured.

### Animals

Adult male C57BL6/J mice (Japan SLC), aged 8–10 weeks, were used in this study. The mice were housed four mice per cage and maintained in temperature- and humidity-controlled specific pathogen-free conditions under a 12 h/12 h-dark/light cycle. All mice were fed a CE-2 diet (CLEA) and tap water *ad libitum*. A stock solution was prepared by dissolving the synthesized ASOs in DNase/RNase-free water. The stock solution was diluted to a volume of 10 μL/1 g mouse body weight for administration according to the following dosage: 1, 3, 10, and 30 mg/kg. The treatment was administered to the mice subcutaneously under isoflurane anesthesia. The administration schedules for each treatment are shown in Figures 2A, 4A, 6A, and 7A. Body weight was measured weekly and urine samples were collected overnight using metabolic cages on the day before euthanasia. All mice were anesthetized and euthanized after overnight fasting, and their whole kidneys were harvested. To establish the IRI model, the left kidney was exposed, and the left renal pedicle was clamped for 25 min. The body temperature of the mice was maintained at a constant 38°C. One week after surgery, a right nephrectomy was performed, and another week later, the mice were sacrificed. This model was used as a positive control for CD45 and KIM-1 staining. All animal care and experimental protocols were approved by the Animal Care and Use Committee of Nagoya University Graduate School of Medicine.

### Biochemical analysis

Biochemical analyses of serum AST, ALT, alkaline phosphatase, total bilirubin, total protein, albumin, creatinine, total cholesterol, low-density lipoprotein cholesterol, high-density lipoprotein cholesterol, triglyceride, uric acid, urinary glucose, urinary protein, and urinary creatinine were performed using an automated chemistry analyzer (Sanritsu Zelkova Laboratory). Urinary NGAL levels were measured using a mouse NGAL assay kit (R&D Systems). Serum IL-6 levels were measured using a mouse IL-6 Quantikine ELISA kit (R&D Systems).

### qPCR

Total RNA from whole mouse kidney samples or HK-2 cells was extracted using an RNeasy Mini Kit (Qiagen), and the RNA concentration was measured using a spectrophotometer (NanoDropLite, Thermo Fisher Scientific). RNA was reverse-transcribed using a cDNA synthesis kit (Qiagen). qPCR was performed with an ABI Step One Plus Real-Time PCR system (Thermo Fisher Scientific) using TaqMan Gene Expression Assays (Applied Biosystems) for mouse *Sglt1*, mouse *Sglt2*, mouse *Tnf-α*, mouse *Tgf-β1*, mouse *F4/80*, mouse *Mcp-1*, and human *SGLT2*. All data were normalized for *β-actin* or glyceraldehyde-3-phosphate dehydrogenase (*GAPDH*) expression.

### Western blotting

Proteins were extracted from renal cortex tissues and cultured cells using radioimmunoprecipitation assay lysis buffer (Santa Cruz Biotechnology) supplemented with 2 mM PMSF, 2 mM sodium orthovanadate, and protease inhibitors. Protein concentration was determined using Pierce BCA Reagent (Thermo Fisher Scientific) according to the manufacturer’s instructions. Western blotting was performed as described previously.^26^^,^^27^ Immunoblotting was performed by incubating the cells overnight at 4°C with anti-rabbit caspase-3 antibody (1:1,000; Cell Signaling Technology), anti-rabbit cleaved caspase-3 antibody (1:1,000; Cell Signaling Technology), anti-rabbit SGLT2 antibody (1:1,000; Proteintech), anti-mouse β-actin antibody (1:10,000; Sigma). The membranes were then incubated with horseradish peroxidase-conjugated anti-rabbit or anti-mouse secondary antibodies for 1 h at room temperature. This was followed by incubation with Peroxidase AffiniPure F(ab’)₂ Fragment Goat Anti-Rabbit IgG, F(ab’)₂ fragment specific (1:5,000; Jackson ImmunoResearch Laboratories) for 1 h at room temperature. The proteins were visualized on an Amersham Imager 600 with an enhanced chemiluminescence detection system (Pierce ECL, Thermo Fisher Scientific), and the intensity of the protein bands was measured using the Amersham Imager 600 analysis software.

### Histological analysis

Harvested murine kidneys and livers fixed in 10% neutral buffered formalin or methyl Carnoy’s solution were embedded in paraffin and cut into 4-μm sections. The sections were then subjected to periodic acid-Schiff, Masson’s trichrome, and hematoxylin and eosin (H&E) staining. For immunofluorescence staining, frozen sections were stained with the following primary antibodies: anti-CD45 antibody (1:20; BioLegend) and anti-KIM-1 antibody (1:200; Bio-Techne, Minneapolis, MN, USA); the sections were subsequently incubated with secondary antibodies Alexa Fluor 555 goat anti-rat IgG (1:1000; Cell Signaling Technology) and Fluorescein goat anti-rat IgG (1:100; Jackson ImmunoResearch Laboratories), respectively. To quantify the CD45^+^ area, eight low-power fields in each section of each mouse were randomly selected and analyzed (each group, *n* = 4). To quantify the KIM-1^+^ area, four low-power fields in each section of each mouse were randomly selected and analyzed (each group, *n* = 4). Digital images were analyzed using HALO image analysis software (Indica Labs).

### *In vivo* imaging and fluorescence microscopy of Cy5-labeled ASO

Cy5-labeled SGLT2-ASOs were subcutaneously administered to mice (detailed protocol is shown in Figure 7A). Various tissues (brain, eye, lung, heart, spleen, kidney, liver, epididymal fat, and small intestine) were harvested and sliced to the same thickness for *ex vivo* imaging. The fluorescence intensity was measured using the IVIS Spectrum system and Living Image Software (PerkinElmer) to evaluate the tissue distribution and accumulation of Cy5-labeled ASOs. The frozen kidney sections were visualized using a Nikon inverted fluorescence microscope (AxioImager M2, Zeiss).

### Statistical analyses

The results are expressed as the mean ± standard error of the mean (SEM). Statistical analyses were performed using one-way ANOVA followed by post hoc Tukey’s multiple comparisons using GraphPad Prism software. *P* <0.05 was considered statistically significant.

## Data and code availability

The data supporting the findings of this study are available from the corresponding author upon reasonable request.

## Acknowledgments

We thank Noriyuki Suzuki, Naoko Asano, Ayako Sakamoto, and Yuriko Sawa for their technical assistance. H.A. and Y.K. received funding from 10.13039/100009619AMED under grant no. 22am0401007. This study was supported in part by Aichi Jinzou Zaidan, Japan. Supports by JP21H05025 (to H.A.) and “Quantum-Based Frontier Research Hub for Industry Development,” 10.13039/501100004823Nagoya University, Japan (to H.A.) are also acknowledged. Supports by 23H04067 (to Y.K.) and Joint Research on ExCELLs (No. 23EXC202) (to Y.K.) are also acknowledged.

## Author contributions

Conceptualization: T.I. and H.A.; writing: T.T., K.H., Y.K., and T.I.; investigation and analyses: T.T., J.A., and K.H.; data curation: T.D., K.I., and J.H.; resources: Y.K. and H.A. (prepared ASOs); supervision: K.F., N.K., Y.I., and S.M.; funding acquisition: S.M. and H.A.; review, editing, & approval of manuscript: all authors. All authors have read and agreed to the final version of the manuscript.

## Declaration of interests

T.T., T.I., Y.K., and H.A. are listed as inventors on a patent application from Nagoya University (WO2021/039598, RNA ACTION INHIBITOR AND USE THEREOF).

## Supplemental information

Document S1. Figures S1–S14

Table S1. Effects of SGLT2-ASOs subcutaneously administered to mice at doses of 10 mg/kg/day thrice per week for three weeks

Data S1. **Target sequence of SGLT2−ASOs**

Document S2. Article plus supplemental information
