# *In vivo* efficacy and safety of systemically administered serinol nucleic acid-modified antisense oligonucleotides in mouse kidney

**DOI:** 10.1016/j.omtn.2025.102497

**Published:** 2025-02-25

**Authors:** Toshiki Tsuboi, Keita Hattori, Takuji Ishimoto, Kentaro Imai, Tomohito Doke, Junichiro Hagita, Jumpei Ariyoshi, Kazuhiro Furuhashi, Noritoshi Kato, Yasuhiko Ito, Yukiko Kamiya, Hiroyuki Asanuma, Shoichi Maruyama

## Main text

(Molecular Therapy: Nucleic Acids *36*, 102387; ▪▪)

In the originally published version of this article, there was an error in Table S1. The authors mistakenly omitted data for urinary glucose. The results for urinary glucose are as follows, and can be found within the revised table:

Urinary glucose (mg/mgCr)(mean ± SEM)

PBS 0.57 ± 0.03

SNA2-ASO 5.54 ± 0.95

MOE-ASO 7.64 ± 1.79

