# Supplementary material for: In vivo efficacy and safety of systemically administered serinol nucleic acid-modified antisense oligonucleotides in mouse kidney
Source: Mol Ther Nucleic Acids. 2024 Dec 18;36(1):102387. doi: 10.1016/j.omtn.2024.102387 (PMC11754010; doi:10.1016/j.omtn.2024.102387)
Supplement: Document S1. Figures S1–S14 [file mmc1.pdf]

OMTN, Volume *36*

Supplemental information

In vivo efficacy and safety of systemically administered serinol nucleic acid-modified antisense oligonucleotides in mouse kidney

Toshiki Tsuboi, Keita Hattori, Takuji Ishimoto, Kentaro Imai, Tomohito Doke, Junichiro Hagita, Jumpei Ariyoshi, Kazuhiro Furuhashi, Noritoshi Kato, Yasuhiko Ito, Yukiko Kamiya, Hiroyuki Asanuma, and Shoichi Maruyama

Figure S1

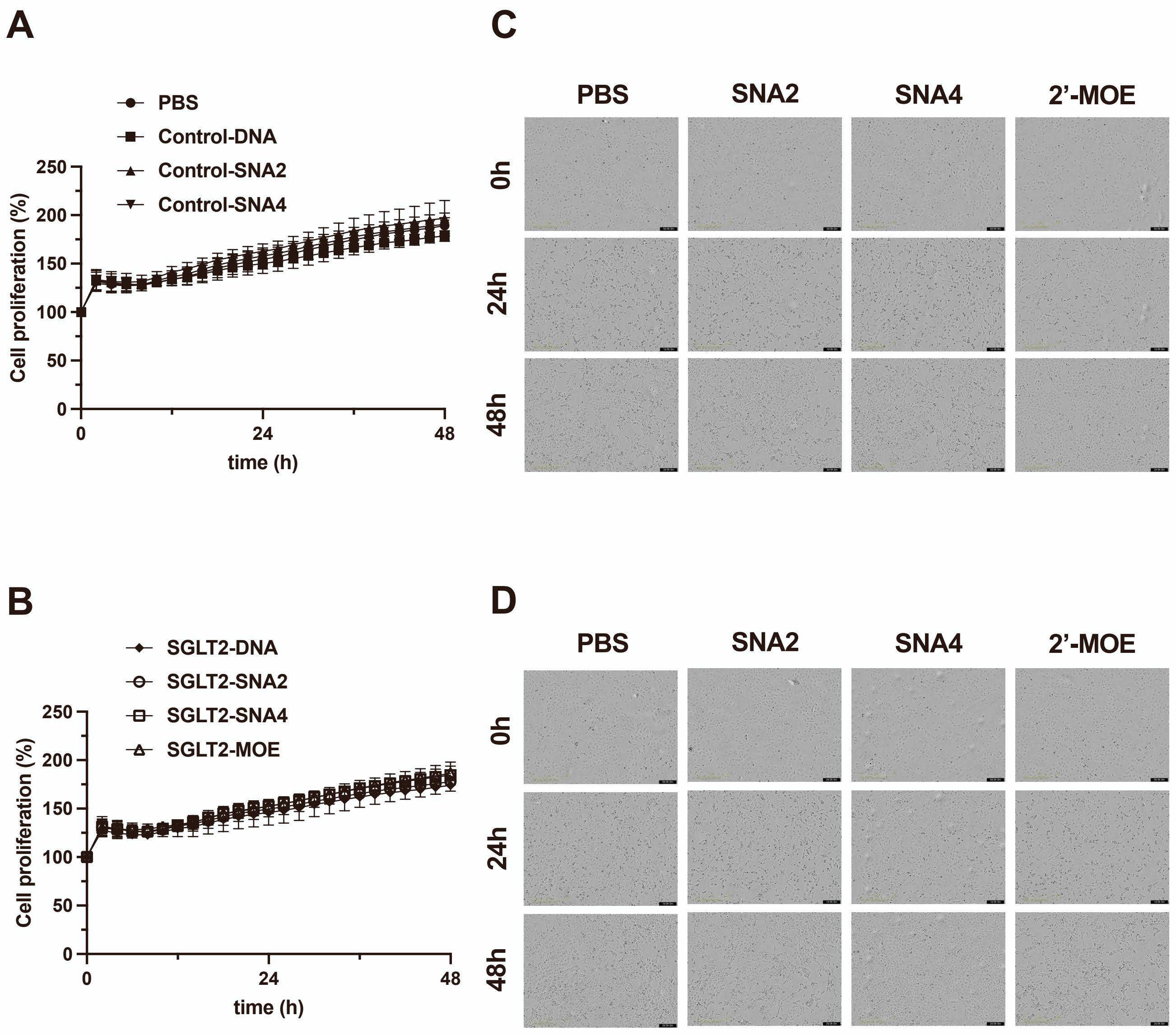

Figure S1.
The HK-2 cells were plated at a density of 1.0×10^5 cells/well onto a 12-well plate and incubated for 24 h. 50 nM of SGLT2-ASOs and control-ASOs were transfected with Lipofectamine 3000. Images were taken every 4 hours until 48 hours, and the cell proliferation rate was measured. (A,B) proliferation ratio (n = 3). Data represent means ± SEM. (C,D) The representative images of adherent cells at 0h, 24h, and 48h for each group. Scale bar = 400 μm.

Figure S2

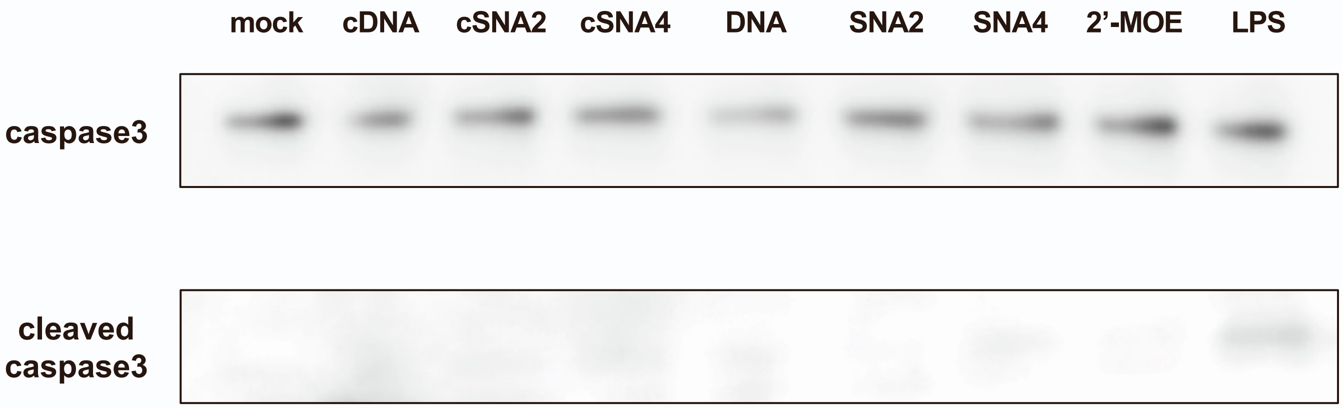

Figure S2.
The HK-2 cells were plated at a density of 5.0×10^5 cells/well in a 6-well plate and incubated for 24 h. 50 nM of SGLT2-ASOs and control-ASOs were transfected with Lipofectamine 3000 and collected after 24 h (n = 3). 10 µg/L of LPS (lipopolysaccharide, Sigma) was used as a positive control. Representative images of western blot of caspase3 and cleaved caspase3.

Figure S3

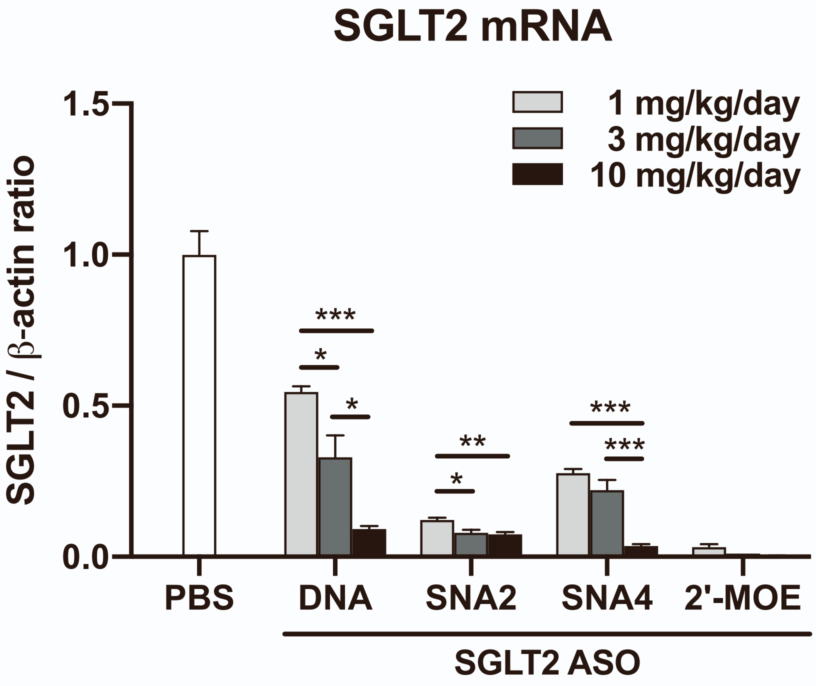

Figure S3

SGLT2-SNA-ASOs suppressed renal SGLT2 expression in a dose-dependent manner SGLT2-ASOs and control-ASOs were subcutaneously (s.c.) administered to mice at doses of 1, 3, and 10 mg/kg/day thrice per week for 1 week. qPCR analysis of SGLT2 expression in the kidney (*n* = 4). β-actin was used as the internal control. Data represent means ± SEM. *P < 0.05, **P < 0.01, ***P < 0.001.

Figure S4

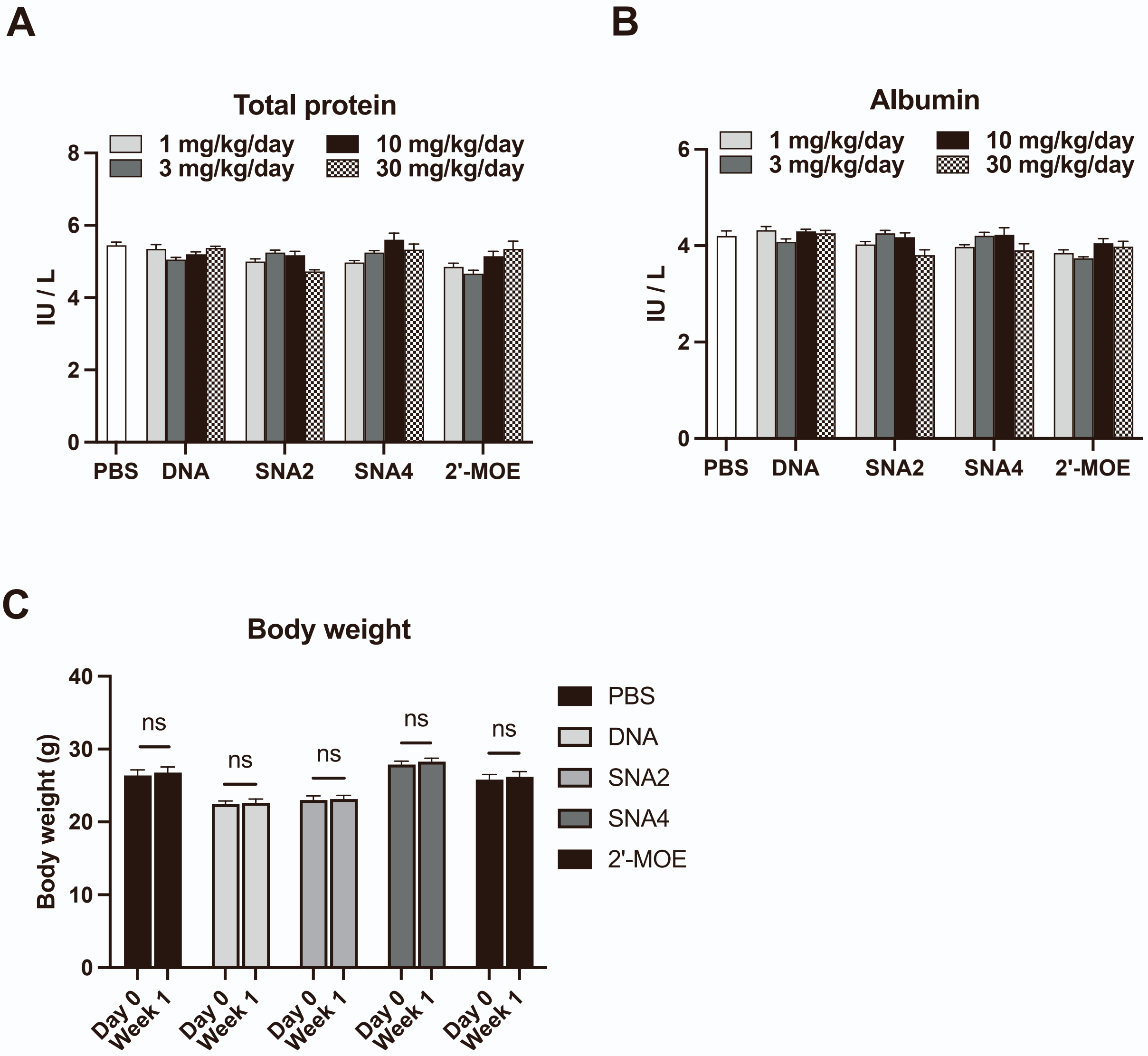

Figure S4.
SGLT2-ASOs were subcutaneously administered to mice at doses of 1, 3, 10, and 30 mg/kg/day thrice per week for 1 week. (A) Serum total protein levels (*n* = 4). (B) Serum albumin levels (n = 4). (C) Body weight of mice administered at doses of 30 mg/kg/day. Data are presented as the means ± SEM. Ns, not significant.

Figure S5

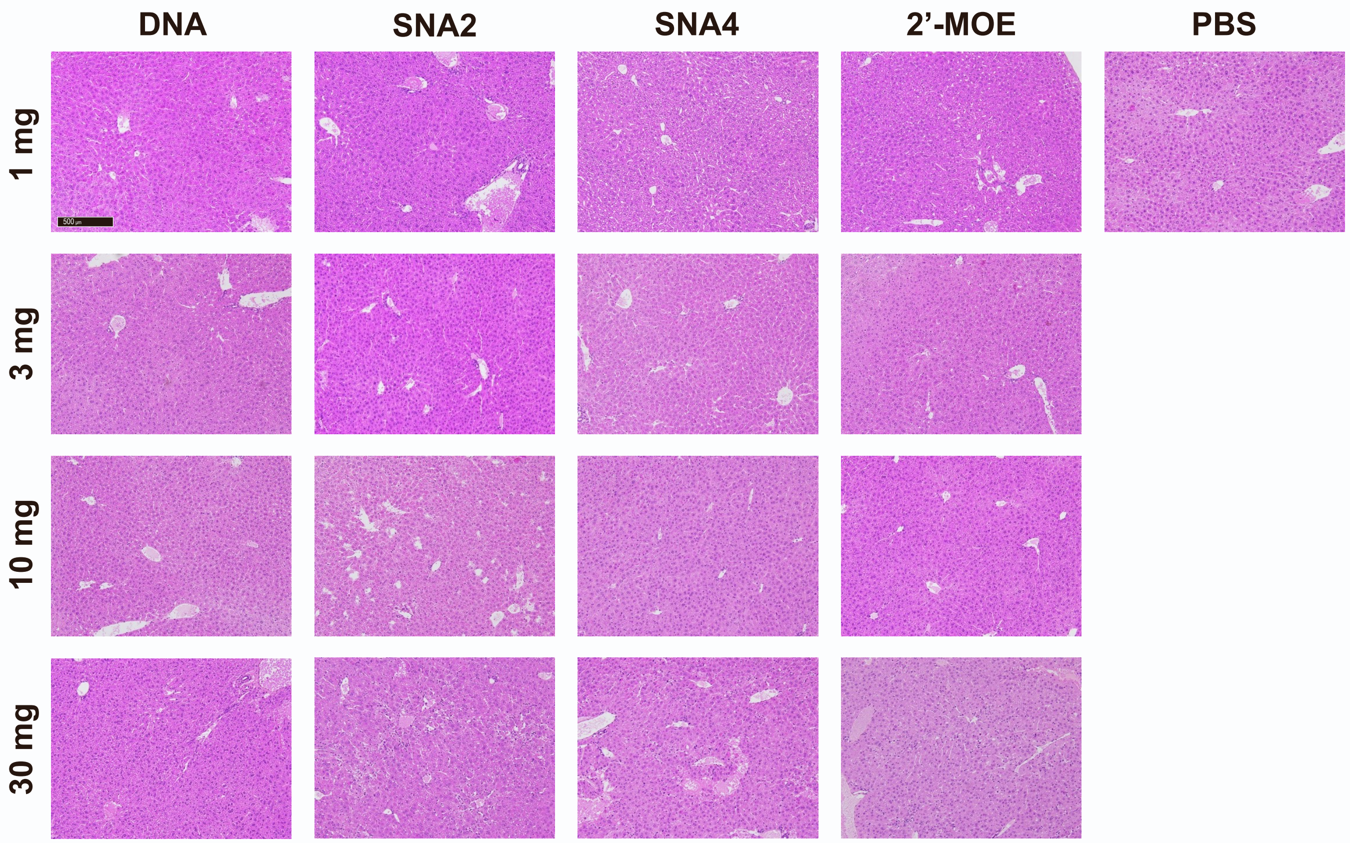

Figure S5. Histopathology of liver (H&E staining)
SGLT2-ASOs were subcutaneously administered to mice at doses of 1, 3, 10, and 30 mg/kg/day thrice per week for 1 week. Representative images of H&E-stained liver. Scale bar = 500 μm.

Figure S6

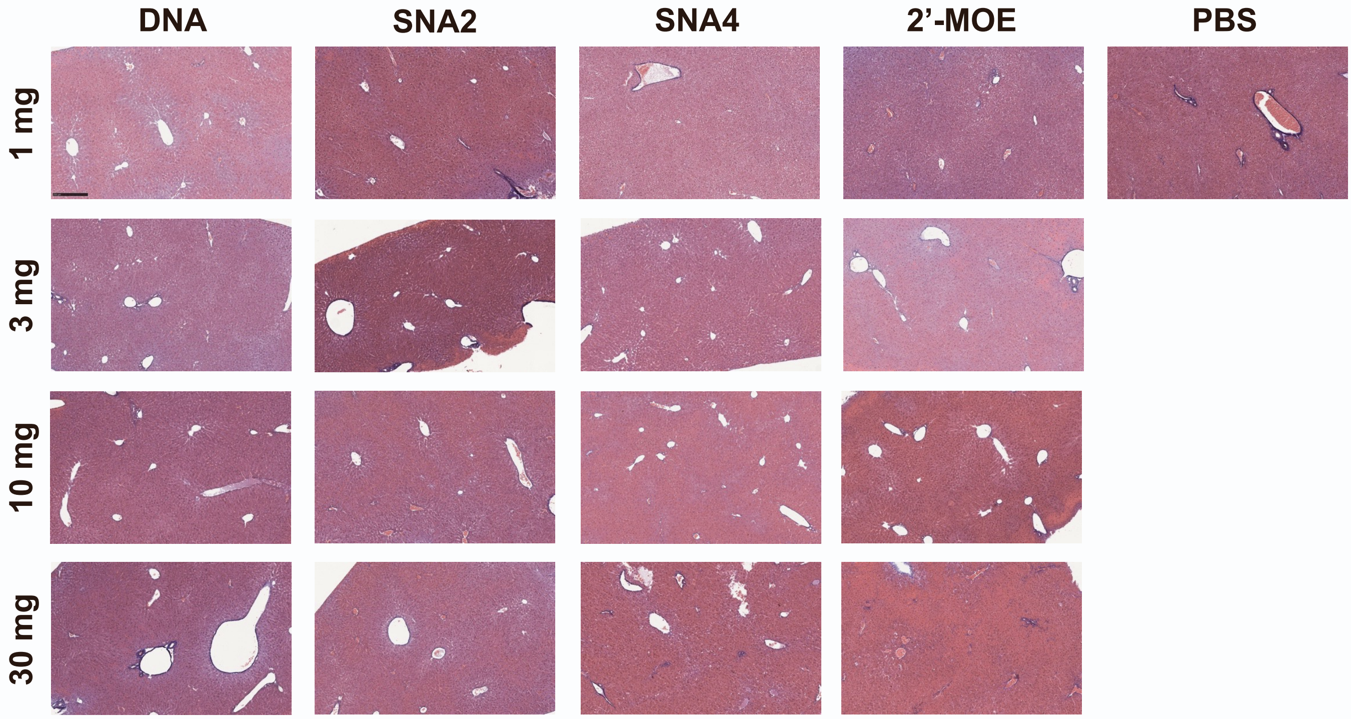

Figure S6. Histopathology of liver (Masson's Trichrome staining)
SGLT2-ASOs were subcutaneously administered to mice at doses of 1, 3, 10, and 30 mg/kg/day thrice per week for 1 week. Representative images of Masson's Trichrome staining of livers. Scale bar = 250 µm.

Figure S7

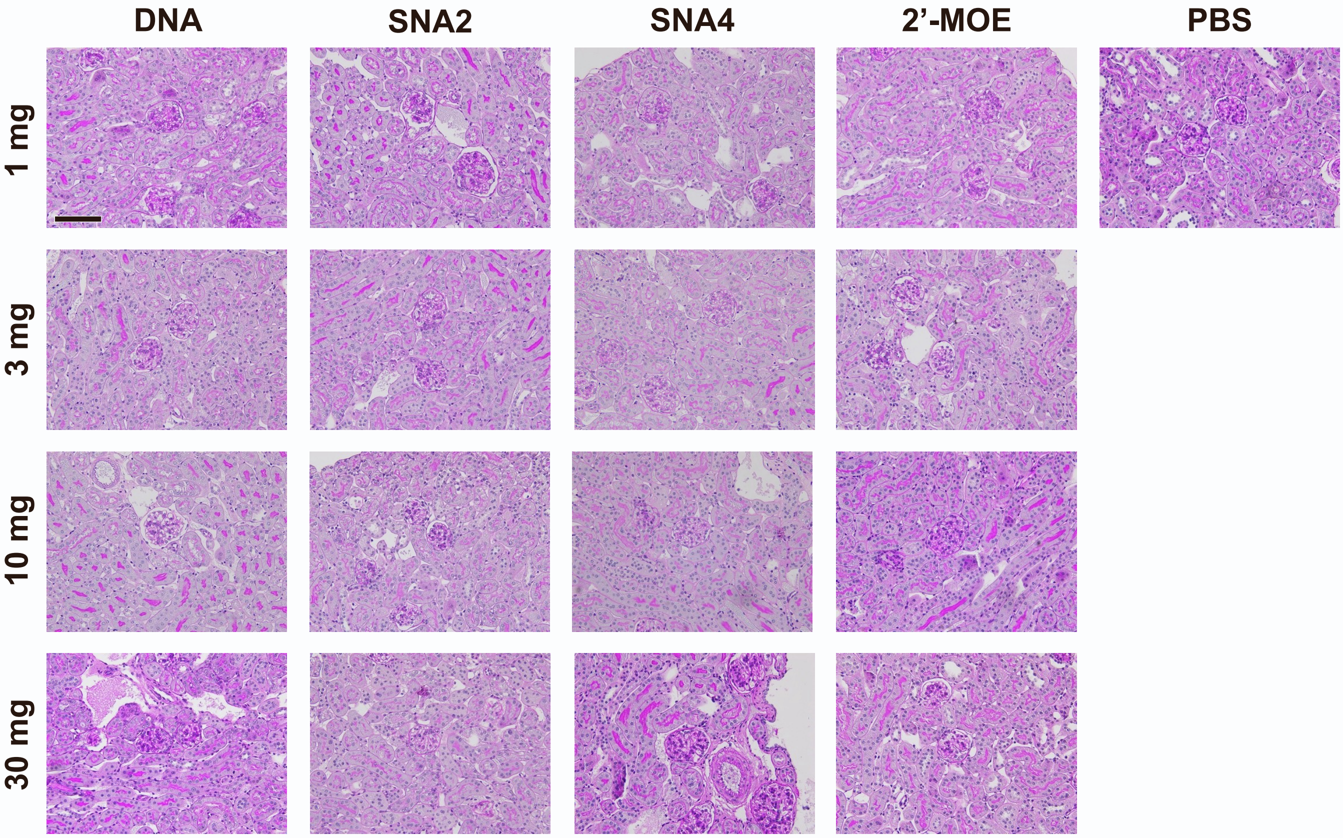

Figure S7. Histopathology of kidney (PAS staining)
SGLT2-ASOs were subcutaneously administered to mice at doses of 1, 3, 10, and 30 mg/kg/day thrice per week for 1 week. Representative images of PAS staining of kidneys. Scale bar = 200 μm.

Figure S8

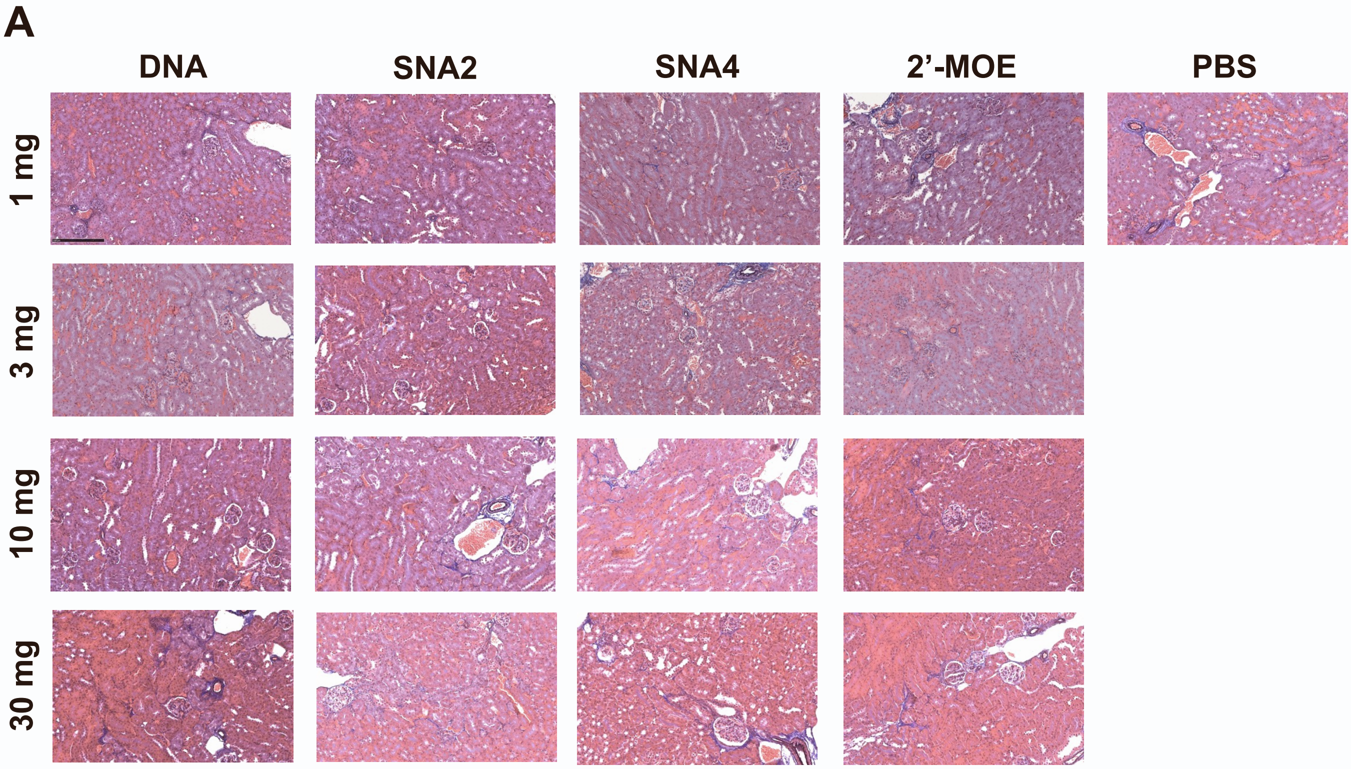

B

	PBS	DNA	SNA2	SNA4	2'-MOE
IFTA score	0.0 ± 0.0	0.3 ± 0.3	0.3 ± 0.3	0.5 ± 0.3	1.0 ± 0.0

Figure S8. Histopathology of kidney (Masson's Trichrome staining)
SGLT2-ASOs were subcutaneously administered to mice at doses of 1, 3, 10, and 30 mg/kg/day thrice per week for 1 week. (A) Representative images of Masson's Trichrome staining of kidneys. Scale bar = 250 µm. (B) IFTA scores for the 30 mg dose group. The results are expressed as the mean ± SEM of 5 to 8 samples each.

Figure S9

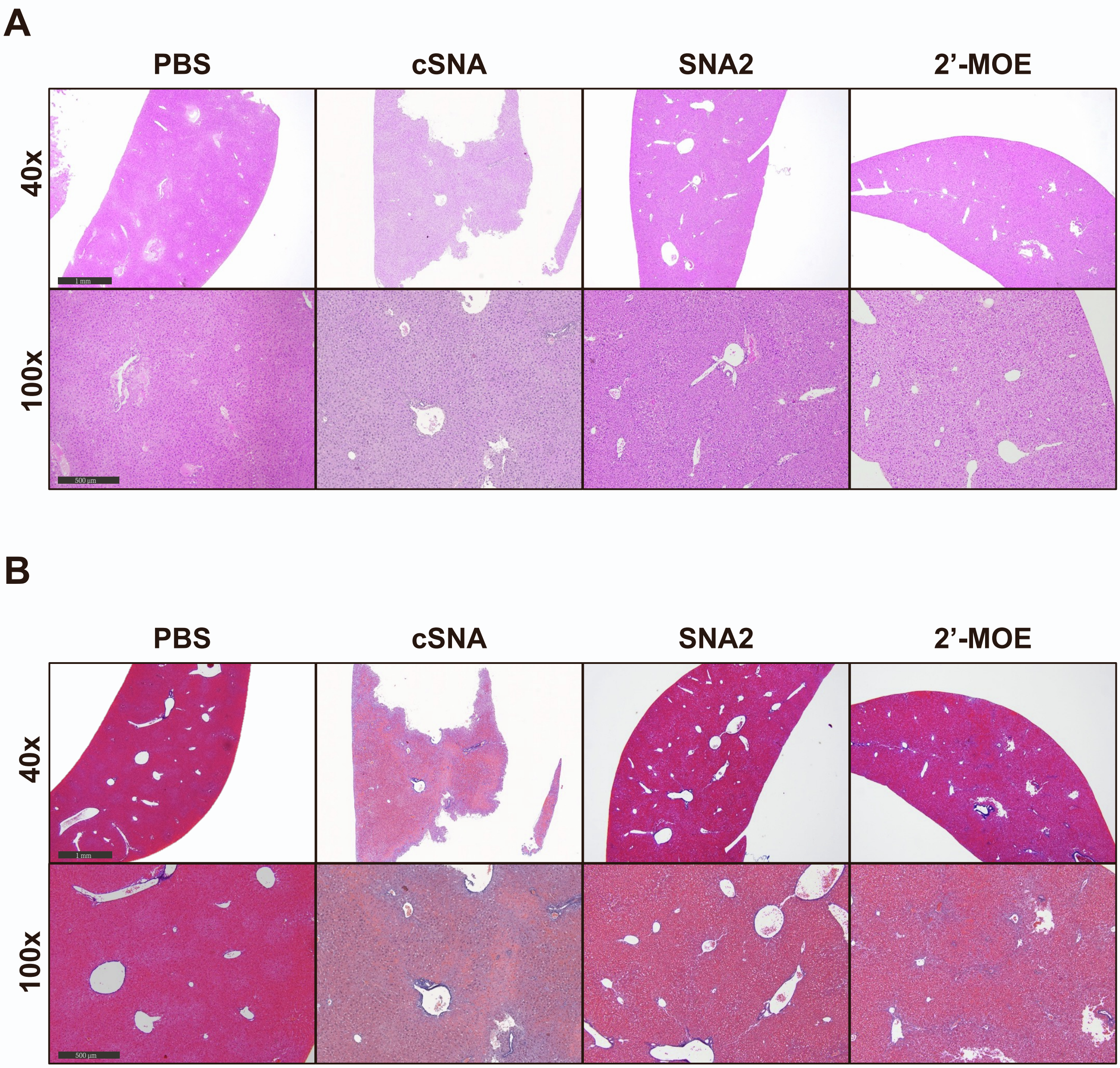

Figure S9. Histopathology of liver

SGLT2-ASOs were subcutaneously administered to mice at doses of 10 mg/kg/day thrice per week for 3 week. (A) Representative images of H&E staining of livers. (B) Representative images of Masson's Trichrome staining of livers. Scale bar = 1 mm for 40x magnification, and 500 µm for 100x magnification.

Figure S10

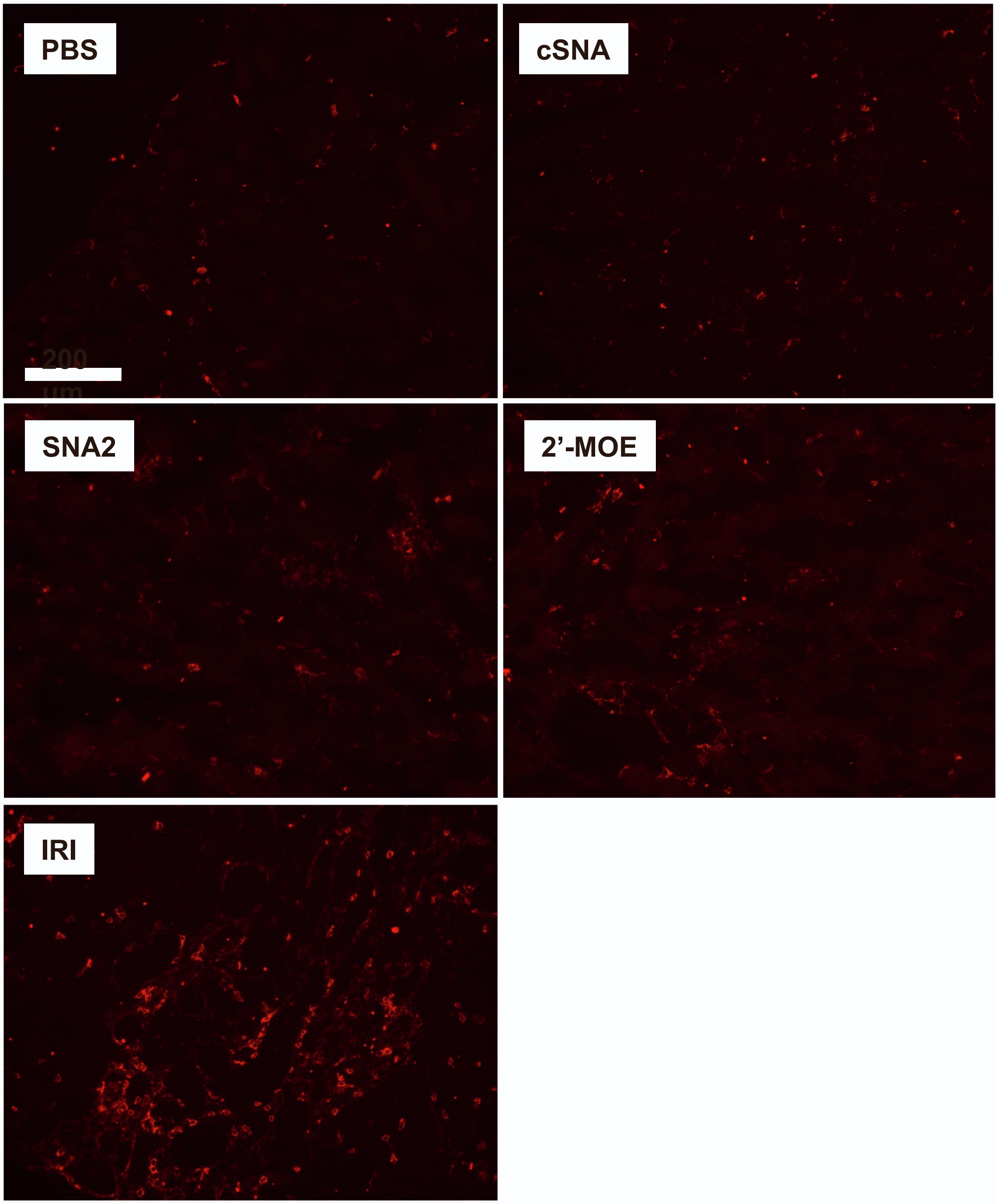

Figure S10. CD45 immunofluorescence staining of kidneys
SGLT2-ASOs, cSNA and vehicle control (PBS) were subcutaneously administered to mice at doses of 10 mg/kg/day thrice per week for 3 week. Representative images of CD45 immunofluorescence staining of kidneys. Kidney specimens from renal ischemia-reperfusion injury (IRI) were used as a positive control for CD45 staining. Scale bar = 200 µm.

Figure S11

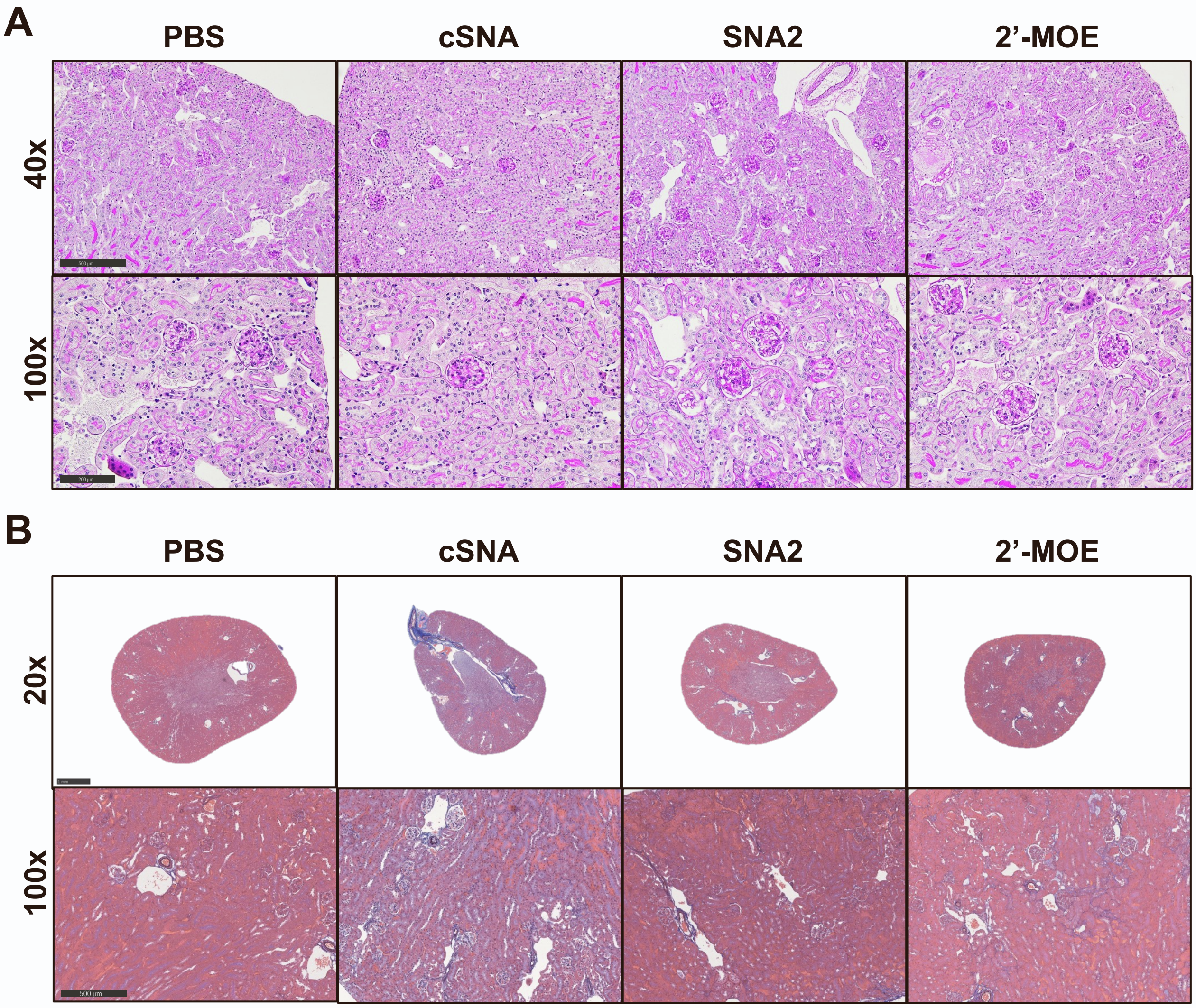

C

	PBS	cSNA2	SNA2	2’-MOE
IFTA score	0.0 ± 0.0	0.0 ± 0.0	0.3 ± 0.3	1.0 ±1.0

Figure S11. Histopathology of kidney

SGLT2-ASOs were subcutaneously administered to mice at doses of 10 mg/kg/day thrice per week for 3 week. (A) Representative images of PAS staining of kidneys. (B) Representative images of Masson‘s Trichrome staining of kidneys. Scale bar = 1 mm, 500 µm, and 200 µm for 20x, 40x, and 100x magnifications respectively. (C) IFTA scores (*n* = 5-8). Data are presented as the means as the means ± SEM.

Figure S12

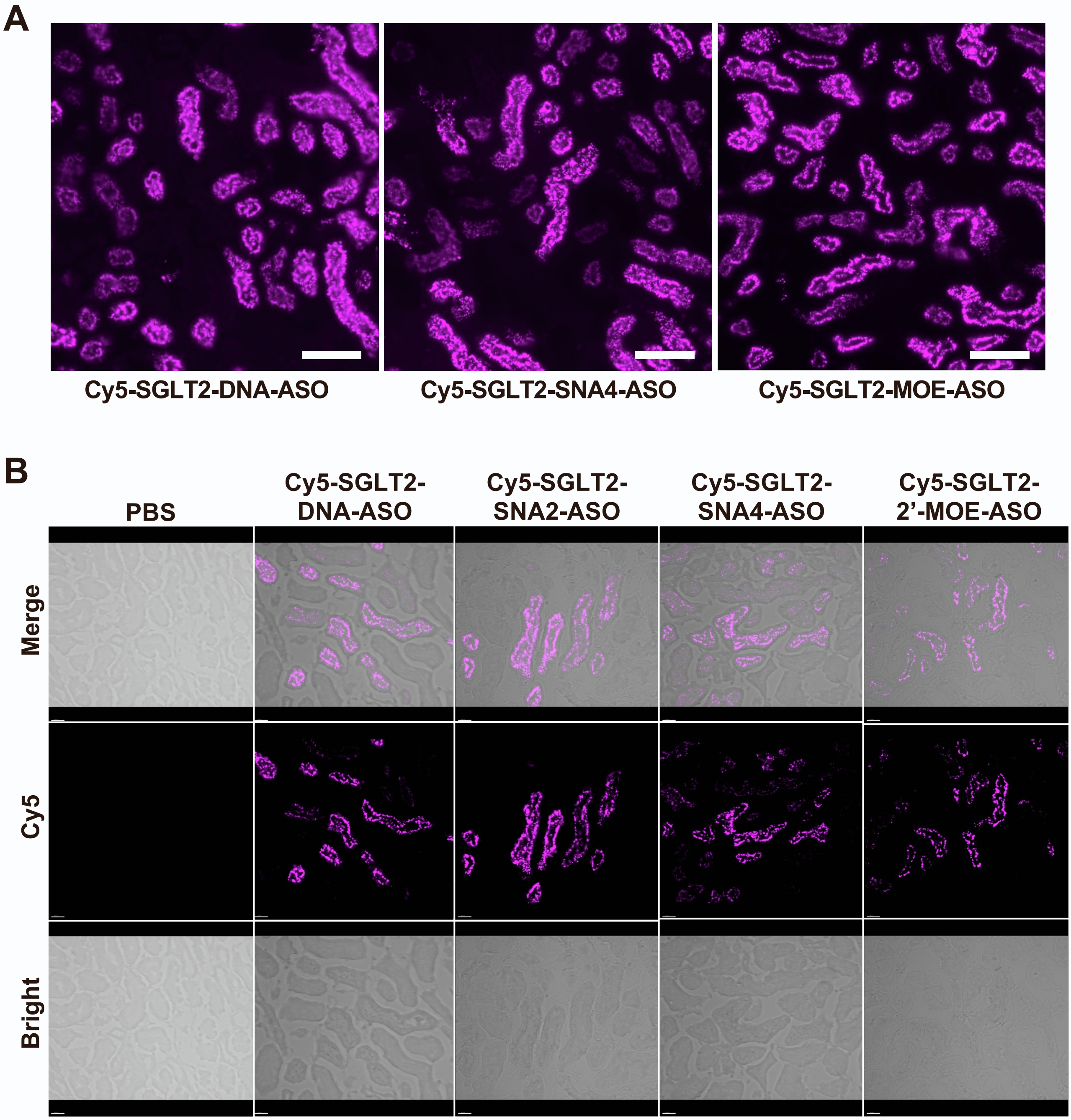

Figure S12. ***in vivo*** **distribution of ASOs in kidneys**

Cy-5 labelled SGLT2-DNA-ASO, SGLT2-SNA2-ASO, SGLT2-SNA4-ASO, SGLT2-MOE-ASO, or PBS (vehicle control) were subcutaneously administered to mice as a single dose of 3 mg/kg. (A) Representative fluorescence microscopy images of kidneys from mice 24 h after the administration of SGLT2-DNA-ASO, SGLT2-SNA4-ASO, and SGLT2-MOE-ASO. Scale bar = 100 μm. (B) Merged images of Differential Interference Contrast (DIC) images and fluorescence microscopy images. Scale bar = 50 μm.

Figure S13

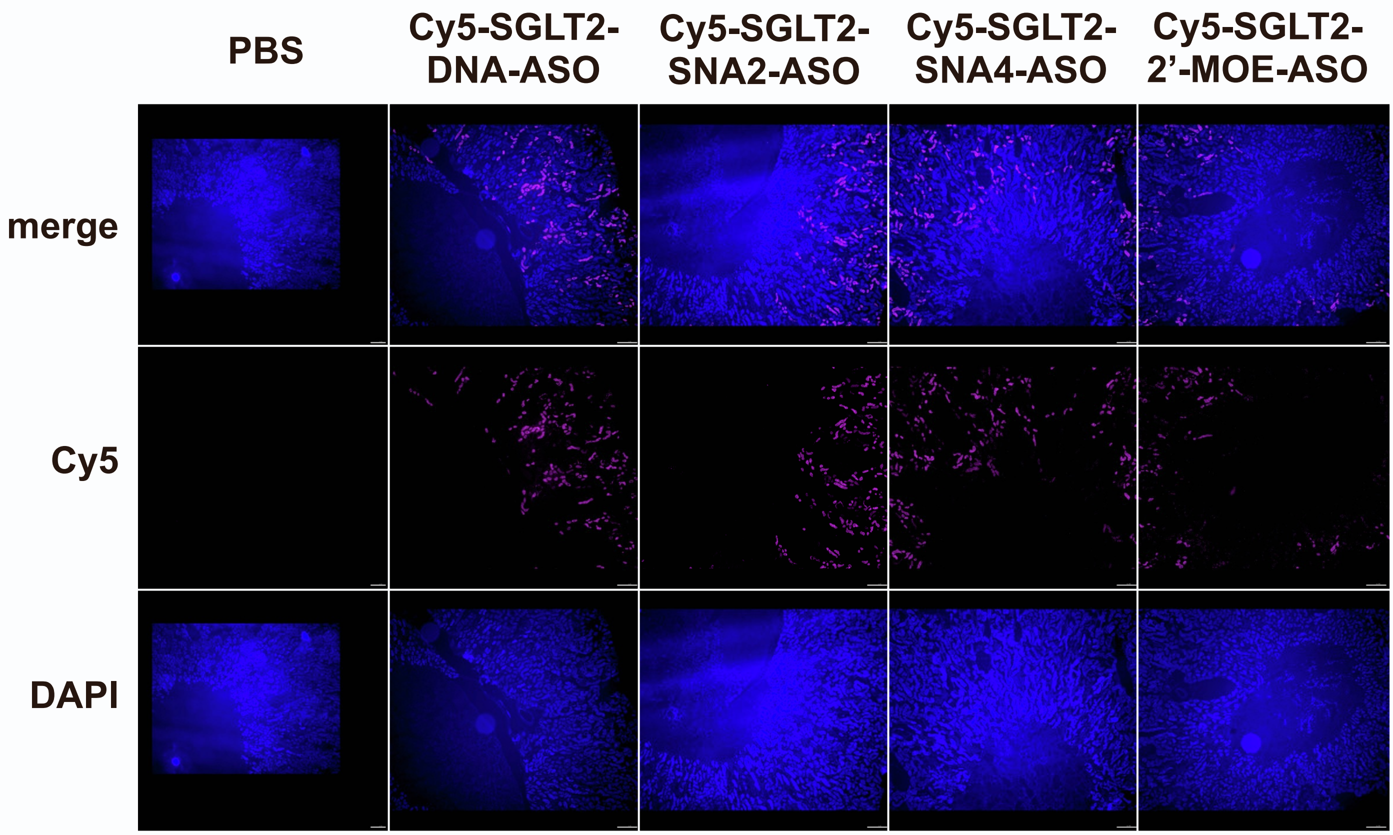

Figure S13. ***in vivo*** **distribution of ASOs in kidneys**

Cy-5 labelled SGLT2-DNA-ASO, SGLT2-SNA2-ASO, SGLT2-SNA4-ASO, SGLT2-MOE-ASO, or PBS (vehicle control) were subcutaneously administered to mice as a single dose of 3 mg/kg. Representative fluorescence microscopy images of kidneys from mice 24 h after the administration of SGLT2-DNA-ASO, SGLT2-SNA4-ASO, and SGLT2-MOE-ASO. Nuclei were stained with DAPI. Cy5 positive area was predominantly observed in the renal cortex (outside of kidney). Scale bar = 50 µm. Scale bar = 400 µm.

Figure S14

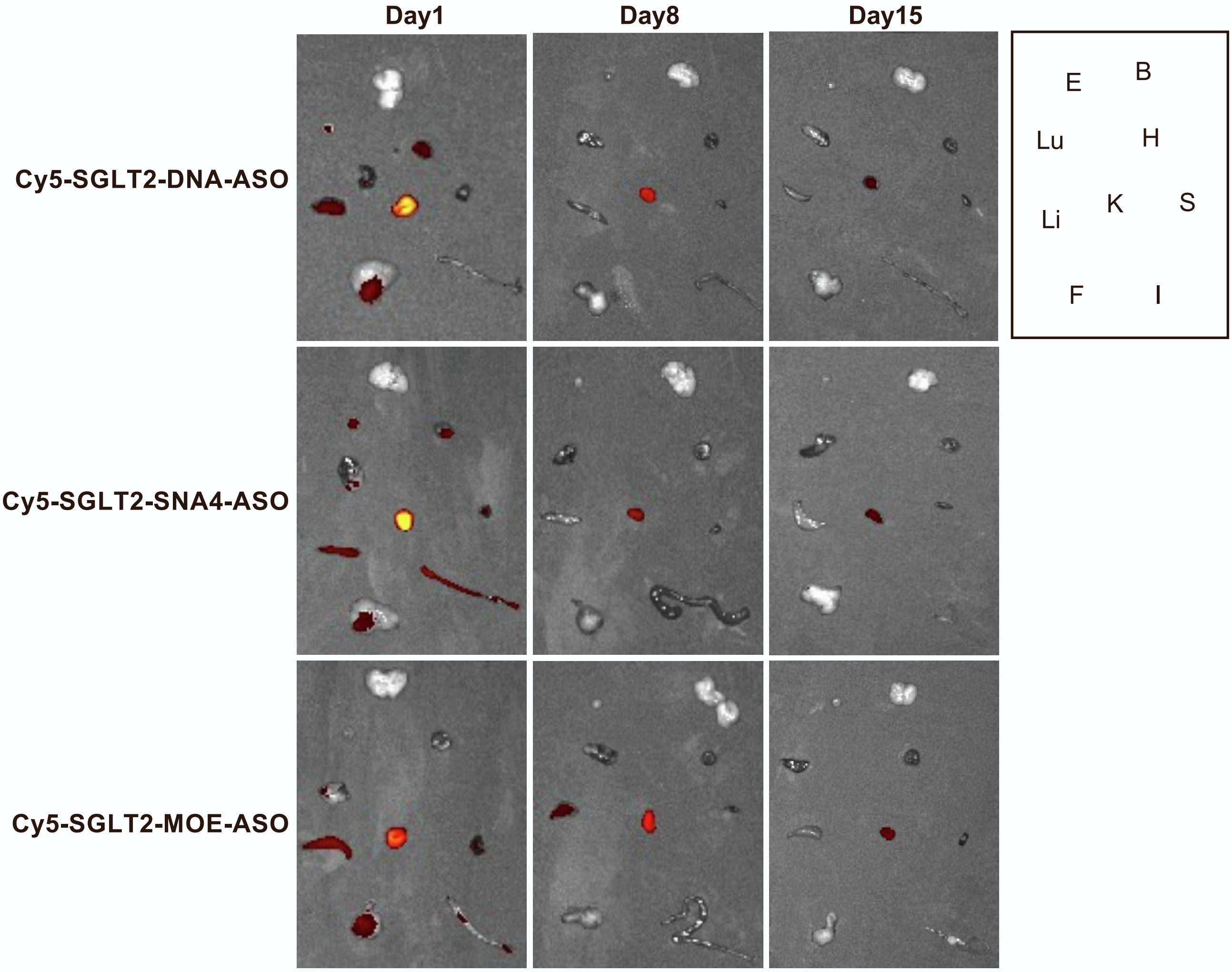

Figure S14. Evaluation of *in vivo* distribution of ASOs

Cy-5 labelled SGLT2-DNA-ASO, SGLT2-SNA2-ASO, SGLT2-SNA4-ASO, SGLT2-MOE-ASO, or PBS (vehicle control) were subcutaneously administered to mice as a single dose of 3 mg/kg. Representative images of fluorescence intensity in each organ, including the kidney (K), liver (Li), brain (B), lung (Lu), heart (H), intestine (I), eye (E), spleen (S), and epididymal fat (F), on days 1, 8, and 15 after ASO administration. Right panel: layout of each tissue.
