# Supplementary material for: In vivo efficacy and safety of systemically administered serinol nucleic acid-modified antisense oligonucleotides in mouse kidney
Source: Mol Ther Nucleic Acids. 2024 Dec 18;36(1):102387. doi: 10.1016/j.omtn.2024.102387 (PMC11754010; doi:10.1016/j.omtn.2024.102387)
Supplement: Table S1. Effects of SGLT2-ASOs subcutaneously administered to mice at doses of 10 mg/kg/day thrice per week for three weeks [file mmc2.pdf]

Table S1.

	PBS (n=8)	SNA2-ASO (n=8)	2'-MOE-ASO (n=8)
Body weight (g)	19.97 ± 0.43	20.47 ± 0.53	19.73 ± 0.23
Kidney (g)	0.0926 ± 0.011	0.0694 ± 0.008	0.0994 ± 0.015
Liver (g)	0.944 ± 0.024	0.944 ± 0.037	0.923 ± 0.012
Heart (g)	0.129 ± 0.005	0.130 ± 0.006$^{###}$	0.105 ± 0.002***
Spleen (g)	0.053 ± 0.002	0.055 ± 0.003$^{\#}$	0.061 ± 0.001**
Creatinine (mg/dL)	0.18 ± 0.013	0.22 ± 0.014	0.20 ± 0.02
Uric acid (mg/dL)	2.15 ± 0.21	1.95 ± 0.16	2.36 ± 0.20
Total-cholesterol (mg/dL)	77.50 ± 3.24	113.88 ± 3.45***	117.88 ± 5.48***
Triglyceride (mg/dL)	24.25 ± 3.69	36.50 ± 5.37	40.63 ± 5.06*
HDL-cholesterol (mg/dL)	51.50 ± 2.31	63.63 ± 2.02***	58.25 ± 1.40*
LDL-cholesterol (mg/dL)	5.00 ± 0.00	8.13 ± 0.63$^{*, ###}$	12.50 ± 1.06***
Urinary protein (mg/mgCr)	9.07 ± 1.71	2.29 ± 0.56***	1.43 ± 0.09***

* P< 0.05, ** P< 0.01, *** P< 0.001 vs PBS. # P< 0.05, ## P< 0.01, ### P< 0.001 vs 2’-MOE.
