# Supplementary material for: In vivo efficacy and safety of systemically administered serinol nucleic acid-modified antisense oligonucleotides in mouse kidney
Source: Mol Ther Nucleic Acids. 2024 Dec 18;36(1):102387. doi: 10.1016/j.omtn.2024.102387 (PMC11754010; doi:10.1016/j.omtn.2024.102387)
Supplement: Data S1. Target sequence of SGLT2−ASOs [file mmc3.pdf]

Data S1

NM_133254.5 Mus musculus solute carrier family 5 (sodium/glucose cotransporter), member 2 (Slc5a2), mRNA

AGATGCTGGAGAGAATGGAGCAACACGTAGAGGCAGGCTCTGAACTTGGGGAGCAGAAGGTCCTGATTGA
TAATCCTGCTGACATTCTGGTTATCGCTGCCTATTTCCTGCTGGTCATTGGTGTTGGCTTGTGGTCTATG
TTCAGAACCAATAGAGGCACAGTTGGTGGCTACTTCCTGGCAGGACGGAGCATGGTGTGGTGGCCGGTTG
GAGCCTCTCTGTTCGCCAGCAACATCGGCAGCGGTCATTTTGTGGGCCTGGCAGGGACTGGCGCAGCAAG
TGGCTTGGCGGTGGCTGGATTTGAGTGGAATGCGCTCTTCGTGGTGCTGCTCCTCGGATGGCTTTTTGTG
CCAGTGTATCTGACCGCTGGTGTGATCACAATGCCTCAGTACCTCCGCAAGCGCTTTGGTGGGCACCGTA
TTCGCCTCTACCTGTCCGTGCTCTCGCTTTTTTTGTACATTTTCACCAAGATCTCGGTGGATATGTTCTC
TGGGGCAGTATTCATTCAACAGGCCCTGGGCTGGAACATTTACGCTTCGGTCATCGCTCTCTTGGGCATC
ACCATGATTTATACTGTGACAGGAGGGCTGGCGGCACTGATGTACACAGACACTGTGCAGACCTTCGTCA
TTCTTGCCGGGGCCTTCATCCTCACTGGTTATGCTTTCCATGAAGTGGGCGGGTACTCGGGTCTCTTCGA
CAAATACCTGGGAGCAATGACTTCACTGACGGTGTCCAAGGATCCATCTGTTGGCAACATCTCCAGCACC
TGCTACCAGCCGAGGCCTGACTCCTATCACCTGCTGCGTGACCCTGTGACAGGAGACCTGCCATGGCCTG
CGCTGCTCCTGGGGCTTACCATTGTCTCGGGCTGGTATTGGTGCAGCGATCAGGTAATAGTGCAGCGGTG
CCTGGCTGGAAAGAATCTGACTCACATCAAAGCTGGGTGCATCTTGTGTGGCTACCTGAAGCTGATGCCC
ATGTTCCTCATGGTCATGCCAGGCATGATCAGCCGCATTCTCTACCCAGATGAGGTGGCATGTGTGGTAC
CTGAGGTGTGTAAGCGGGTGTGTGGCACTGAGGTGGGCTGCTCTAACATCGCCTACCCACGGCTCGTGGT
GAAGCTCATGCCCAATGGTCTGCGCGGACTCATGCTGGCAGTCATGCTGGCTGCCCTCATGTCTTCTCTG
GCATCCATCTTTAACAGCAGTAGCACGCTCTTCACCATGGATATCTACACGCGCCTGCGGCCCCGTGCAG
GTGATAAGGAGCTGCTGCTAGTTGGAAGGCTCTGGGTGGTATTCATCGTGGCGGTGTCCGTGGCTTGGCT
GCCAGTGGTGCAGGCAGCTCAGGGTGGGCAGCTCTTCGATTACATTCAGTCTGTCTCCAGCTATCTGGCA
CCTCCAGTGTCTGCGGTCTTTGTGCTTGCACTCTTTGTGCCCCGTGTTAATGAGAAGGGAGCCTTCTGGG
GACTAGTTGGGGGCCTGCTGATGGGCCTAGCTCGTCTCATACCCGAGTTCTTCTTTGGCTCGGGCAGCTG
TGTGCGACCCTCAGCGTGCCCGGCACTCTTCTGTCGGGTACACTACCTCTATTTCGCCATCATTCTCTTC
ATCTGCTCTGGCATCCTCACACTTGGAATCTCCCTGTGCACTGCGCCCATCCCTCAGAAGCATCTCCATC
GCCTGGTTTTCAGTCTCCGGCACAGCAAGGAGGAGCGGGAGGACCTGGATGCTGATGAGTTAGAAGGTCC
AGCCCCTGCTCCTGTGCAGAACGGGGGCCAGGAATGTGCAATGGAGATGGAAGAGGTCCAGTCCCCGGCT
CCAGGCCTGCTCCGCCGGTGCCTGCTTTGGTTCTGTGGGATGAGCAAGAGTGGGTCAGGGAGTCCTCCGC
CCACTACCGAGGAGGTGGCGGCAACCACCAGGCGGCTGGAGGACATCAGTGAGGATCCCCGCTGGGCACG
AGTAGTCAACCTCAATGCCCTACTCATGATGACCGTGGCTGTGTTCCTCTGGGGCTTCTATGCATAAAGT
CGAGGGTGTTGGATGCCATGAGCTACAACCAGGCCATGTTGGACCCTCACAAAGAGTAAGGGTGAGCAGC
TTGGAGTGGATCCCAGAAAAGGAACAGGGCAAGAATACAGCAGGAAGGAACCGGTTCCCTTCCTCTTTAC
CCGGGGTCCAGTCCATTTGATTGGTTGTCACTTCCCACAAGATGATGGCCAATTGGTCATAGAGGTTTGC
CTATACAAAAATAAAACTGCCCTCCTAACA

Bold and underlined, the target sequence of SGLT2−ASOs
